# Insights into Toxic *Prymnesium parvum* Blooms as a Cause of the Ecological Disaster on the Odra River

**DOI:** 10.3390/toxins15060403

**Published:** 2023-06-19

**Authors:** Janusz Sobieraj, Dominik Metelski

**Affiliations:** 1 Department of Building Engineering, Warsaw University of Technology, 00-637 Warsaw, Poland; janusz.sobieraj@pw.edu.pl; 2 Research Group SEJ-609 “AMIKO”, Faculty of Economics and Management Sciences, Campus de Cartuja s/n, University of Granada, 18071 Granada, Spain

**Keywords:** harmful algal blooms, ecological catastrophe, prymnesins, river ecosystem, aquatic toxicity, water pollution, environmental monitoring, eutrophication, microalgal ecology, fish kill

## Abstract

In 2022, Poland and Germany experienced a prolonged and extensive mass fish kill in the Odra River. During the period from the end of July to the beginning of September 2022, a high level of incidental disease and mortality was observed in various fish species (dozens of different species were found dead). The fish mortality affected five Polish provinces (Silesia, Opole, Lower Silesia, Lubuskie, and Western Pomerania) and involved reservoir systems covering most of the river (the Odra River is 854 km long, of which 742 km are in Poland). Fatal cases were investigated using toxicological, anatomopathological, and histopathological tests. Water samples were collected to determine nutrient status in the water column, phytoplankton biomass, and community composition. High nutrient concentrations indicated high phytoplankton productivity, with favorable conditions for golden algal blooms. The harmful toxins (prymnesins secreted by *Prymnesium parvum* habitats) had not been found in Poland before, but it was only a matter of time, especially in the Odra River, whose waters are permanently saline and still used for navigation. The observed fish mortality resulted in a 50% decrease in the fish population in the river and affected mainly cold-blooded species. Histopathological examinations of fish showed acute damage to the most perfused organs (gills, spleen, kidneys). The disruption to hematopoietic processes and damage to the gills were due to the action of hemolytic toxins (prymnesins). An evaluation of the collected hydrological, meteorological, biological, and physico-chemical data on the observed spatio-temporal course of the catastrophe, as well as the detection of three compounds from the group of B-type prymnesins in the analyzed material (the presence of prymnesins was confirmed using an analysis of the fragmentation spectrum and the accurate tandem mass spectrometry (MS/MS) measurement, in combination with high-performance liquid chromatography-tandem mass spectrometry (LC-MS/MS), allowed the formulation and subsequent testing of the hypothesis for a direct link between the observed fish mortality and the presence of prymnesins in the Odra River. This article systematizes what is known about the causes of the fish kill in the Odra River in 2022, based on official government reports (one Polish and one German) and the EU technical report by the Joint Research Centre. A review and critical analysis of government findings (Polish and German) on this disaster were conducted in the context of what is known to date about similar cases of mass fish kills.

## 1. Introduction

Localized fish kills, which can range from a few individuals to millions of dead fish (including the extinction of entire fish populations), are visible signs of environmental stress [1] and can lead to the death/disappearance of aquatic life [2]. Such events are usually investigated urgently by environmental agencies to determine the causes [3]. Fish die-offs are important indicators of aquatic environmental problems, and although some fish species are very sensitive to adverse changes in environmental conditions (there are many fish species that have a relatively low tolerance to changes in the environment) [4,5,6], it is important to remember that such changes usually affect entire aquatic ecosystems, and thus other animals and plants, as well as everything related to the bottom life of the aquatic environment [7]. Localized sudden and mass fish kills or even whole fish populations and deterioration (mortality) in aquatic life in different types of water bodies, namely freshwater, marine, and estuarine, have been observed quite frequently and excessively in recent years [3,8]. Although the causes of their occurrence may be natural, anthropogenic changes and pollution (including toxins) in aquatic and terrestrial systems are major contributors to the increasing frequency and magnitude of fish kills worldwide [3,9]. Many ecological events and disasters that have led to mass fish kills are related to anthropogenic activities, such as pollution from agricultural runoff or biotoxins [10]. Industrial disasters around the world have also caused significant health, environmental, and economic damage [11].

The effects of a mass fish kill have negative consequences for the local communities associated with the affected areas (although this is, of course, the case in most cases) and are frequently the cause of enormous economic losses for entire countries where such events occur [12]. This is because mass fish kills lead to significant declines in tourism, recreation, and commercial activities in affected areas [13,14]. Fish kills can also negatively impact food security, such as drinking water production [15]. In addition, there are cleanup costs and other measurable losses. For example, according to a Polish report (on the impact of the environmental disaster in the Odra River), 249 tons of dead fish were collected in the Odra River from July to August 2022 (in the period from late July to 12 September 2022, in a total of five provinces) [16]. In addition, Holmlund and Hammer [13] point out the negative prospect of changes in food web dynamics and nutrient balance resulting in less fish protein available for human consumption [13], and Koehn [17] points out the costs of long-term fishery closures. As a result of anthropogenic activities that discharge wastewater, detergents, and fertilizers—pollutants containing nitrates or phosphates—into the water system, more and more industrial and municipal pollutants and toxins are entering rivers and oceans. This type of pollution contains large amounts of nitrogen and phosphorus compounds (so-called biogenic substances), the presence of which is the cause of the phenomenon known as eutrophication [18], i.e., the so-called overfertilization of water by a large number of nutrients (under such conditions, there is an excessive accumulation of minerals and nutrients in the water) and massive algal and toxic cyanobacterial blooms [19]. Over time, the algae decompose, which is accompanied by a decrease in oxygen levels in the deeper layers of the water [20], which in turn leads to the proliferation of anaerobic bacteria responsible for the production of hydrogen sulfide [21]. As a result of eutrophication, so-called “oxygen deserts” are formed [22], which significantly complicate conditions for organisms that need oxygen to live.

The total ecological, social, and economic damage caused by the ecological disaster in the Odra River (in the context of the very high number of fish killed) shows the serious scale of the disaster, which, of course, translates into measurable economic damage to fisheries, tourism, food web dynamics, and aquatic ecosystems. This makes it all the more important to systematize the causes of fish kills associated with this disaster and to engage in a broader discussion of the causes in order to develop and implement appropriate preventive measures to avoid such incidents in the future. Specifically, this article deals with (1) the analysis of the hydrometeorological situation in the period before the fish kill; (2) the analysis of the water quality in the Odra River during the period before the fish kill; (3) toxicological and anatomical–pathological studies on the fish; (4) the identification of the presence and bloom of *P. parvum*; (5) the identification of the presence of PKS genes; (6) the determination of the prymnesins produced by *P. parvum*; and (7) the analysis of the satellite images. It provides a systematization of what is known about the causes of the fish kill in the Odra River (in Poland) from July to September 2022. This article draws on two government reports (one Polish and one German) that set out, among other things, the government’s findings about the disaster, and reviews and summarizes these reports. Government researchers who studied this ecological disaster concluded that all the conditions were in place for a massive bloom of an invasive, non-native microalgae species (*P. parvum*). Moreover, there is a serious risk that a mass bloom of *P. parvum* could be repeated in the Odra River. Therefore, it is worthwhile to consolidate knowledge about the methods used in other countries of the world to control *P. parvum*. The structure of this study is very simple. Section 2 discusses toxins and toxin-producing microalgae, emphasizing that they are the main cause of fish kills. Section 3 contains a detailed presentation of the results presented in official government reports (both Polish and German). Section 4 contains a detailed discussion followed by the conclusions in Section 5.

## 2. Toxins and Toxins Secreted by Algal Bloom Habitats—Major Causes of Fish Kills

### 2.1. Toxins

Toxins can pose a lethal threat to aquatic fish for several reasons They can, for example, (1) damage gills and other organs, making it difficult for fish to breathe and survive; (2) disrupt the nervous system, leading to disorientation, paralysis, and death [23]; (3) damage the liver, kidneys, and other organs, leading to organ failure and death [24]; (4) disrupt the hormonal system, leading to reproductive failure [25,26,27]; (5) disrupt the immune system and make fish more susceptible to disease [28,29]; (6) impair fish growth and development, leading to abnormal growth or even death [30]; and (7) accumulate in fish tissues and can be passed through the food chain, making fish unsafe for human consumption [31]. It is important to know that toxic substances can come from many sources, including industrial pollution, agricultural runoff [32,33], and even naturally occurring toxins produced by certain species of algae or bacteria [34,35]. It is important to monitor water bodies and take steps to reduce or eliminate sources of toxins to protect fish populations and the ecosystem as a whole. The following are some examples of specific toxins that can contribute to ecological disasters and the mass mortality of fish: (1) Ammonia—a toxic compound that can enter water bodies through agricultural runoff [36], industrial effluents, and other forms of pollution. High levels of ammonia can impair the respiratory capacity of fish and lead to mass fish kills [37,38]. (2) Heavy metals such as lead, mercury [39,40], and cadmium can be toxic to fish, and high concentrations of these compounds can cause mass fish kills [41]. Heavy metals can enter waterways through industrial wastewater, agricultural runoff, and other forms of pollution. (3) Polychlorinated biphenyls (PCBs)—a group of chemicals that were widely used in industry and are now banned in most countries. PCBs can remain in the environment for decades and can be toxic to fish [42], leading to massive fish die-offs [43,44,45]. (4) Cyanide, which can also be toxic to fish and leads to massive fish kills [46]. Cyanide can enter waterways through industrial wastewater, agricultural runoff, and other forms of pollution. (5) Oil spills, which can cause massive fish kills by smothering fish and disrupting oxygen levels in the water [47,48]. (6) Pesticides—toxic to fish and cause massive fish kills [49]. Pesticides can enter waterways through agricultural runoff and other forms of pollution [50]. (7) Algal blooms—some harmful algal blooms can produce toxins that can be lethal to fish and other aquatic life and cause massive fish kills [14,51,52]. It is important to note that the toxins that cause mass fish kills can vary by location and pollution source. It is important to investigate and understand the cause of fish kills to prevent future incidents and protect the aquatic ecosystem. 

There are numerous examples in the literature of mass deaths of fish caused by the presence of toxins in the aquatic environment. Most often, such cases occur in connection with the discharge of substances into water bodies that are directly toxic or cause a change in the pH of the water or its temperature. Harmful substances that enter water bodies (aquatic environments) as a result of anthropogenic activities may themselves be toxic (cause toxicity) or cause a change in the properties of the water, such as its pH or temperature, which may subsequently lead to mass fish kills. Larson et al. [53] described fish kills in the United States caused by pesticides, particularly endrin. Researchers refer to numerous cases of fish kills caused by the mixing zone effect [54], i.e., harmful interactions between acid, calcium, and labile aluminum at certain concentrations [55] or chemical interactions with complex polymeric aluminum salts [56]. Rosseland et al. [54] described in detail the phenomenon of mixing zones and chemical imbalances caused by transformation processes. Such mixing zones occur near acidic tributaries and stream limestones. For example, it was the effect of the mixing zone that was responsible for the near extinction of the perch population in the L. Iso Valkjaervi watershed in the early 1990s. Supersaturated aluminum solutions with their persistent active precipitates proved to be particularly toxic to fish. This phenomenon was confirmed with experiments in a mixing zone in the limed Audna River in Norway. Rosseland et al. [54] studied the stress to which Atlantic salmon and sea trout were subjected by mixing acidic and calcareous water downstream of the crossing. In the acidic tributary, the LT50 (a term used in toxicological studies that refers to the median lethal time, i.e., the time that elapses before 50% of a test population dies after exposure to a toxic substance or stress condition; it is commonly used to quantify the amount of a stressor necessary to kill an organism) was 22–40 h (depending on the fish species), while in the mixing zone (different pH parameters, Ca in mg/L and Ali), LT50 was 7 h for both species. Thus, due to the conversion of Al into high-molecular-weight precipitation species, failure of osmoregulation and lethal changes in fish gills occurred in the mixing zone. The results indicate greater toxicity of the mixing zones on the health status of fish than in acidic and Al-rich water.

It is important to emphasize that cases of toxic conditions are more likely in poorly buffered water. Accidental discharges of various chemicals, such as acidic process water, are not uncommon. One example is the 1997 discharge of acidic process water into Skinned Sapling Creek that accidentally occurred at a phosphate plant in Mulberry, Florida. At that time, tens of millions of gallons of acidic water were discharged into Skinned Sapling Creek, significantly lowering the pH of the water (from 8 to 4) [57] and contributing to the mass mortality of more than one million fish [57,58]. The discharge of acidic process water from a phosphate plant could be caused by a failure in the plant’s safety systems, such as a leak in the retention pond or a malfunction in the treatment process. This type of discharge can have significant environmental impacts, such as the death of fish and other aquatic life, and can also have economic impacts on local communities that depend on fishing and tourism. Acidic industrial process water can contain a variety of toxins, including heavy metals and other pollutants, depending on the specific industrial process. These toxins can be harmful to both human health and the environment if not properly managed. In addition, acidic industrial process water can also cause environmental damage if discharged into natural waters. It is important to know that acidic industrial process water should be treated before it is discharged into the environment. Treatment options include neutralization to raise pH, precipitation, and flocculation to remove heavy metals and other pollutants, and biological treatment to remove organic pollutants. The specific treatment options depend on the type and concentration of contaminants present in the process water [59]. It is also important to know that regulations for the treatment and discharge of industrial process water vary by region and industry. Therefore, it is important to contact the appropriate authorities to ensure compliance with all applicable regulations. Other cases include an accidental spill of bourbon whiskey into the Kentucky River in 2000 [60]. The incident occurred when Wild Turkey’s whiskey warehouse caught fire. At that time, several thousand barrels of burning whiskey spilled into the surrounding area, with about 20% of the whiskey entering the Kentucky River, resulting in a reduction in oxygen levels in the water and a mass mortality of about 228,000 fish in a 66-mile stretch of the river. In 1999, a fish kill in the White River in Indiana was caused by a chemical spill. The cause of the fish kill was initially unknown, but ammonia contamination was suspected [61]. The investigation traced back to Guide Corp, a manufacturer of automotive lamps that had discharged toxic waste into the river [62]. In 2011, the Temple-Inland paper mill in Bogalusa, Louisiana, discharged chemicals into the Pearl River, causing a massive fish kill [63]. In 1995, a 120,000-square-foot lagoon at Oceanview Farms in North Carolina burst and discharged twenty-five million gallons of fecal matter and sewage into the New River [64]. This killed at least ten million fish and polluted 350,000 acres of coastal shellfish habitat. A fish kill in Michigan’s Tittabawassee River in 2020 was caused by pollution from a toxic waste dump (as a result of the Edenville Dam collapse) [65]. In 2023, hazardous materials were released into the Tittabawassee River and transported downstream to the Saginaw River and Bay [66]. In the past, there have been even more serious ecological disasters involving fish kills associated with the Tittabawassee River. In 1986, up to 30 million gallons of diluted chemicals entered the Tittabawassee River, resulting in fish contamination (tainting) [67]. The incident had significant environmental impacts, including the death of hundreds of thousands of fish. According to another source, an industrial spill in the mid-1960s killed an estimated 14,000 fish in the Tittabawassee River near Midland [68]. In 2017, PFAS contamination of drinking water and fish was discovered in the Huron River in southeast Michigan [69]. Fish kills can also be caused by toxins produced by harmful algae. There are many species of harmful algae, and one of them is *P. parvum*, which is cited as the main cause of the 2022 disaster in the Odra River, which is analyzed in more detail in this article. Therefore, this particular species of algae will be discussed in more detail in Section 2.2 and subsequent sections. 

### 2.2. Algae Blooms and Harmful Toxins Secreted by Habitats of Algal Blooms

There are several types of harmful algal blooms that can release toxins that can be lethal to fish. The most common types of harmful algal blooms include red tide (*Karenia brevis*) [70,71], cyanobacterial blooms [72,73], brown tide (*Aureococcus anophagefferens*) [74], diatom blooms [75], and *P. parvum* (also known as golden alga) [76,77,78]. These blooms can produce different types of toxins that can cause various symptoms in humans and animals. It is widely recognized that there are more toxic algal species today than in the past, which has led to higher economic losses and impacts on fisheries resources [79]. Algal blooms occur when nutrient concentrations in the water are elevated, due to the discharge of wastes and fertilizers (or other chemicals) into the water. Heisler et al. [80] note that degradation in water quality due to increasing nutrient pollution promotes the development and persistence of many harmful algal blooms. The Centers for Disease Control and Prevention (CDC) explain that harmful algal blooms usually form in warm waters with high levels of nutrients such as nitrogen and phosphorus [81]. The US Environmental Protection Agency (EPA) and National Oceanic and Atmospheric Administration (NOAA) also state that harmful algal blooms require nutrients (nitrogen and phosphorus) to form [82,83]. It is worth noting that not all species of algae should be associated with the secretion of toxins that are harmful to fish (only some). Algae are dangerous to fish because—when they die and decompose—they lead to a reduction in the oxygen content of the water (deprive it of oxygen), which under the right conditions (high temperature, too low or too high pH) can be the cause of mass fish death. Very often, mass mortality of fish is attributed to a combination of several of the above causes (higher temperatures, low water levels, and algal blooms). This was the case, for example, in Lake Peipsi in Estonia in the summer of 2002, where massive fish kills were caused by the synergistic effect of high temperature, low water levels, and a blue–green algal bloom [84].

**Figure 1:**
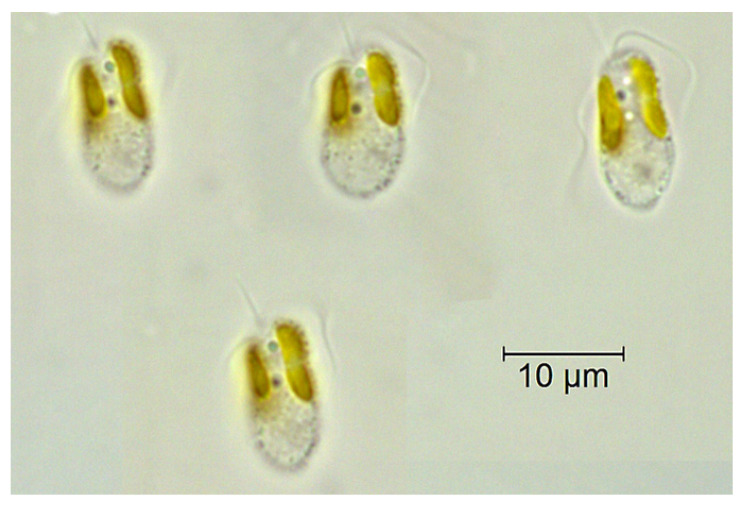
*P. parvum* (N. Carter 1937) [85].

The first mention of algal blooms associated with *P. parvum* (indicated as the causative agent of the 2022 Odra disaster) was published by Liebert and Deerns in 1920 [78]. However, it should be noted that a detailed description of *P. parvum* based on specimens from a brackish pond at Bembridge on the Isle of Wight was published by Carter in 1937 [85]. Moreover, the prymnesins were first isolated only in the late 20th century (1995 to be precise) [86]. Relevant comments on the systematic classification (arrangement) of haptophytes, taking into account their taxonomy at the species and morphological levels, can be found in the work of Larsen [87] and Edvardsen et al. [88]. Furthermore, *P. parvum* (N. Carter) was well-documented and described in the 1960s by Manton and Leedale [89] and Manton [90]. *P. parvum* is a species of haptophyte, a group of microalgae characterized by the possession of haptonemes, slender whip-like appendages that aid in motility. The cells of *P. parvum* are typically 3.5–11 µm wide and 6–18.5 µm long [91,92] (see Figure 1). 

*P. parvum* is a species of golden alga that has flagella and chloroplasts for photosynthesis [93]. It has a complex life cycle that includes both sexual and vegetative phases. This microalgae species is known for its coccolith production and its ability to produce a toxic compound that is harmful to other aquatic animals. It can form blooms in freshwater and marine systems and negatively impact the ecosystem and water quality. According to Roelke and Manning [76], the life cycle of *P. parvum* has three stages: two haploid stages, one diploid stage, and a survival stage called a cyst. 

There is extensive literature describing cases of *P. parvum*. This article focuses on the analysis of *P. parvum* and the harmful toxins prymnesins secreted by these algae, which are considered to be the direct cause of the environmental disaster in the Odra River in 2022. Therefore, selected cases that occurred in different countries are briefly characterized in Table 1.

## 3. Summary Report

### 3.1. Geographical Location of the Ecological Disaster in the Odra River

**Figure 2:**
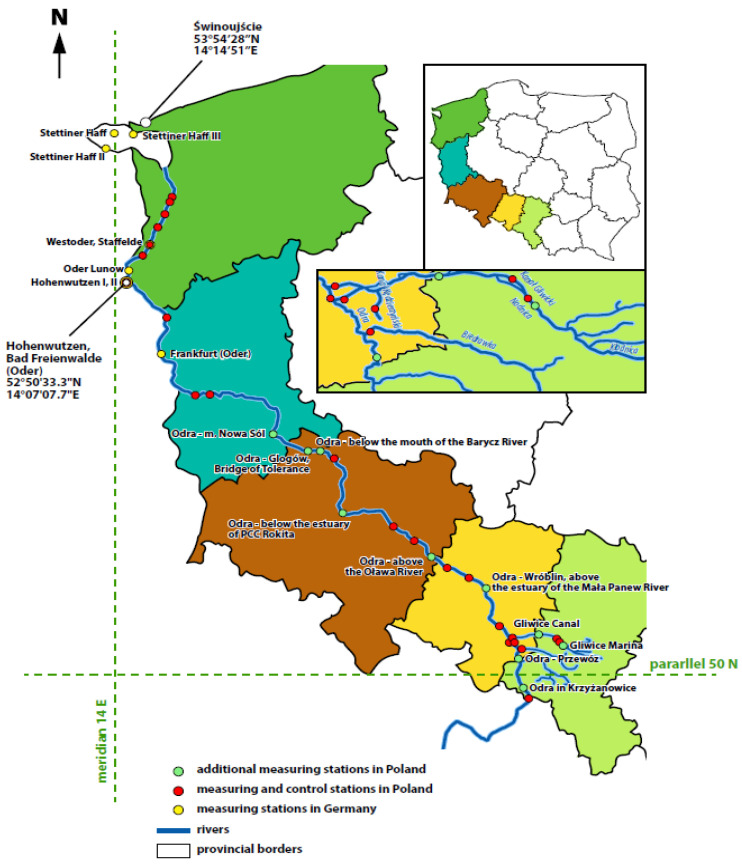
River Odra—geographical position with indication of measuring and control stations. Note: Different colors indicate 5 different Polish voivodeships (provinces) through which the Odra River flows (order from the top): West Pomeranian Voivodeship, Lubuskie Voivodeship, Lower Silesian Voivodeship, Opole Voivodeship, and Silesian Voivodeship. (source: own elaboration).

First of all, although fish kills are quite common and occur in many places in the world, there are not too many summaries or reports explaining the possible causes of their occurrence (at regional or national level) [3,107]. Therefore, it is to be welcomed that both Polish and the German research groups (each side conducted separate, independent investigations) tackled this difficult task of investigating the causes of this environmental disaster of enormous scale at a rapid pace, resulting in the publication of preliminary reports on the causes of this disaster at the end of September 2022 (more precisely, both sides published separate reports on 30 September 2022).The result of the disaster in the Odra River was a fish kill on a scale never before recorded in that river. The fish kill was documented at locations in five Polish provinces: Silesia, Opole, Lower Silesia, Lubuskie, and Western Pomerania, i.e., practically along the entire length of the river. It should be recalled that the Odra River rises in the Silesian Beskids in the Czech Republic at the coordinates 50.2067° N, 18.9317° E and flows through five different Polish provinces, forming part of the border between Poland and Germany before flowing into the Baltic Sea via an estuary. The approximate coordinates for the mouth of the Odra River into the Baltic Sea are 54.4759° N, 14.5753° E (see Figure 2).

### 3.2. Polish Report Summarizing the State of Knowledge about the Environmental Disaster in the Odra River

The Polish report summarizing the state of knowledge about the environmental disaster in the Odra River that occurred in July and August 2022 was published in late September 2022 [16]. Numerous government agencies were involved in the preparation of the report, including the General Directorate for Environmental Protection (GDOŚ), the General Inspectorate for Environmental Protection (GIOŚ), the Institute of Meteorology and Water Management—National Research Institute (IMiGW), the State Veterinary Institute, the Veterinary Inspection, the Institute of Inland Fisheries (IRŚ), the State Water Holding Company “Polish Waters”, and numerous scientific centers, including the Warsaw University of Technology, the Universities of Warsaw, Wroclaw, Gdansk, Olsztyn, and Vienna, the Universities of Life Sciences in Wroclaw and Lublin, and the Wrocław University of Technology.

**Figure 3:**
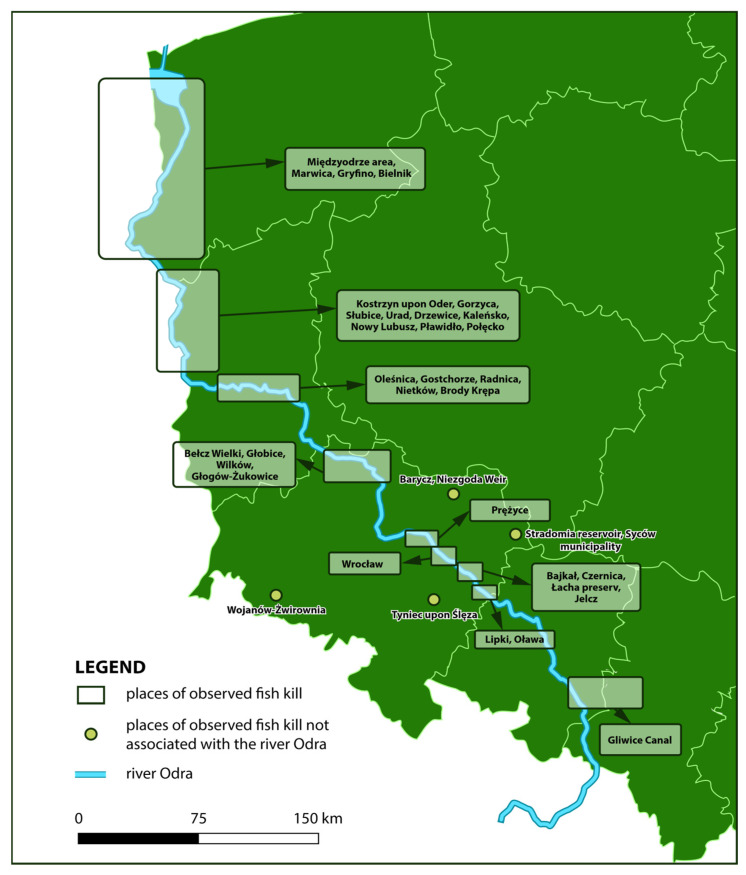
Locations along the Odra River where fish kills were reported (source: own elaboration).

According to the report, 249 tons of dead fish were documented along the Odra River in July and August 2022, but these data can be considered estimates. Figure 3 shows locations along the river where fish kills were reported.

The error for underestimation was not stated, and with few exceptions, e.g., data collected by the Regional Directorate for Environmental Protection (RDOŚ), estimates for specific fish categories were not provided (the exception being the very sketchy data provided by the Polish Anglers Association). Significantly, the State Water Holding Company “Polish Waters”, which has been criticized for numerous failures in the management of its waters [108,109], did not provide any data [16]. As for the catastrophe, the fish kill was not a uniform phenomenon, that is, it was not observed in all sections of the river. It is important to point out that almost no data were collected on the extent of damage to animal organisms associated with the bottom of the water body (the so-called benthos), but it should be emphasized that in addition to fish, mollusk mortality was also reported. It turns out that the meteorological and hydrological conditions (in July and August 2022) in which the environmental disaster occurred were not extreme. According to what the hydrographs from this period show, the levels/volumes of the discharges were in line with the values of the average low water of many years (they stacked up well below the average). In some river sections, instantaneous values of discharges fluctuated within the limits of the historically lowest low flows (e.g., below Malczyce, 40 km from Wroclaw in the northwestern direction). During the study period, there were only a few intensive rainfalls in the Odra River basin, which led to a periodical increase in discharges, which then reached the values of average discharges and in some places, even higher discharges.

As for the water quality of the Odra River (against the background of the ecological disaster), multi-year measurements along the entire length of the river (before the event) showed a poor state of physico-chemical and biological elements and a poorer chemical state. The high conductivity and high chloride, sulfate, and magnesium levels may indicate very unfavorable (unacceptable) physico-chemical environmental conditions. In contrast, the inadequate biological condition (condition of biological elements) was due to the condition of the benthic macroinvertebrate and fish communities. The chemical condition, rated less than good, was the result of pollutants in the water—heptachlor, brominated diphenyl ethers, fluoranthene and benzo(a)pyrene, and mercury and its compounds in the biota. Although the analyzes revealed a higher than average conductivity and salinity along the entire length of the Odra River (highest in the tributaries at the level of the Kłodnica and Gliwice canals), it could not be proven that the increase in salinity led to this disaster, because the investigations, including the integration and systematization of the methods used, were carried out too late (practically several weeks after the outbreak of the disaster, when the wave of fish mortality had already reached the lower Odra River).

On the Polish side, the highest conductivities were measured at the beginning of August 2022 (at the start of the measurement period), with values that were in some cases well above 2000 μS/cm and locally even reached values averaging >5000 μS/cm, with a maximum of >7000 μS/cm in Kłodnica and the Gliwice Canal). In comparison, on the German side, the timing of fish mortality coincided with the increase in conductivity, and the highest conductivities (>2000 μS/cm) were measured in the period between 7 and 14 August 2022 at the Frankfurt (Oder) monitoring station. Significantly, the accepted conductivity standards for a river such as the Odra are 700–850 μS/cm (the upper end of this range are typical values for a large lowland river). However, it should be kept in mind that the standards were still exceeded in the years before the environmental disaster. The average values in the years before were between 1030 and 1287 μS/cm (in Wroclaw). In any case, it is important to emphasize that the period of fish death coincided with a significant increase in conductivity.

The Polish report also includes the results from analyses of satellite images showing chlorophyll profiles in different sections of the Odra River waters, as well as toxicological, anatomopathological, and histopathological examinations of dead fish. Chlorophyll analyses based on Sentinel-2 satellite imagery (within the Copernicus program, in which the European Space Agency (ESA) uses the Sentinel-1 and Sentinel-2 satellites) showed elevated chlorophyll concentrations in the Gliwice Channel, Lake Turawa (east of Opole), and Lakes Paczkowski, Otmuchowski, and Nyski west of Nysa (near the Czech border). Despite the observation of a migrating chlorophyll wave in the Odra River, no algal bloom was detected in the Żelazny Most Reservoir.

The results of toxicological examinations of dead fish (based on samples taken on 12 August 2022—these were the first samples) did not show above-average pollution, i.e., values that would differ from those typical for Polish rivers (recorded in previous measurement periods); however, it should be emphasized that the observed values exceeded the norms for permissible levels of mercury and organic compounds (although this has often occurred in the past). Therefore, it is not possible to conclusively demonstrate that the exceedance of standards in this regard is the result of pollution attributable to the July–August 2022 environmental disaster because it could just as easily be the result of fish living in a polluted/contaminated environment for many years. Histopathological and anatomical studies conducted by Polish researchers indicated damage to the gills, kidneys, and spleen of the fish. The researchers suspected that hemolytic toxin is the probable cause of this damage. Based on the evidence collected by the Polish researchers, the algal bloom of *P. parvum* and the prymnesin toxins secreted by this alga were identified as the most likely cause of fish deaths. This research hypothesis was confirmed using the results of samples taken from the water of the Odra River, especially from the lagoons connected to the Odra River and the Gliwice Canal, in which large amounts of *P. parvum* and the prymnesins secreted by this alga were detected. The circumstantial evidence, especially the temporal and spatial sequence, suggests that a local proliferation of large amounts of *P. parvum* algae occurred in the waters of the upper Odra River. However, it was difficult to determine the exact location where the possible propagation of *P. parvum* may have occurred. Normally, the reproduction of phytoplankton does not take place in the rivers themselves but in dams, on various obstacles, or in reservoirs on the river banks, and later, they enter the river itself. A whole series of different studies (conducted by the Polish side) point to a number of causes of the disaster, which together contributed to this massive environmental catastrophe. The main factors mentioned in the report are increased conductivity, increased water temperature, increased solar radiation, chloride and sulfate content, significant fluctuations in water parameters over time, and hydromorphological changes in the river, which led to a slowdown in the river in many places (in weirs, channels, etc.), which in fact created the right conditions for toxic algal blooms. In addition, Mazur-Marzec et al. [110] determined the prymnesins (PRMs) produced by *P. parvum*. Using fragmentation spectra (more specifically, a QTRAP5500 mass spectrometer) and accurate measurements (HRMS QTOF mass spectrometer, Bruker), these authors demonstrated the presence of ichthyotoxins of the B-type prymnesins group in the samples studied. They also investigated the relationship between the number of cells of *P. parvum* and the concentration of prymnesins (semi-quantitatively determined) in the studied sites, which proved to be statistically significant. Similarly, a correlation between the presence of *P. parvum* in the water and fish mortality in the middle reaches of the Odra River was demonstrated. Details on the method used by Mazur-Marzec et al. [110] to determine the prymnesins (PRMs) produced by *P. parvum* are described in Section 3.2.1.

#### 3.2.1. Identification of Prymnesins in the Odra River 

The toxic effect of the haptophyte *P. parvum* on fish and invertebrates is due to the presence of prymnesins, which are polyketides with a polycyclic ether structure [111]. According to Binzer et al. [112], there are at least three types of prymnesins—A, B, and C—that have a similar skeleton but have different toxicity due to differences in structure, such as the substitution of sugar residues or the presence of chlorine atoms. In areas comparable to Poland, only group B prymnesins have been identified. The detection of prymnesins is usually performed using high-resolution tandem mass spectrometry (MS/HRMS); however, high-performance liquid chromatography (LC-MS/MS) can also be used. The choice of the method depends on various factors such as the sensitivity, selectivity, and resolution required for the analysis [113,114]. However, since there is no commercially available certified standard, quantitative analysis can only be performed using indirect methods and requires access to a standard for validation. From 19 August to 9 September 2022, the Institute of Inland Fisheries in Olsztyn sent 260 seepage pit samples and lysophilized mussels to the Department of Marine Biotechnology at the University of Gdansk for analysis. Of these samples, 231 filter tubes were subjected to extraction and LC-MS/MS analysis, while the remaining 28 filter cells were subjected to extraction only. The samples were sent to the Department of Food Chemistry and Toxicology, University of Vienna, for the determination of toxin concentrations using the indirect method described in Svenssen et al. [115] and Medić et al. [116]. Material collected from the area affected by the *P. parvum* bloom was subjected to extraction and LC-MS/MS analysis to determine the presence and concentration of prymnesin toxins. The extraction process, as described by Binzer et al. [112], involved the use of cold acetone and 100% methanol to remove and extract the toxins, respectively. The resulting methanol extract was analyzed using a QTRAP5500 spectrometer in an untargeted, information-dependent analysis mode. This allowed for a general ion scan and fragmentation of the compounds in the sample, leading to the determination of the elements of their structure. A high-resolution mass spectrometer (HRMS QTOF, Bruker) was also used to further confirm the structure of the compound. The analyses revealed the presence of at least three prymnesin B toxins in the samples from the *P. parvum* bloom area, as indicated by the presence of doubly charged ions [M+2H]^2+^ and pseudomolecular ions [M+H]^+^ as well as sodium adducts [M+Na+H]^2+^ (Table 2). 

**Figure 4:**
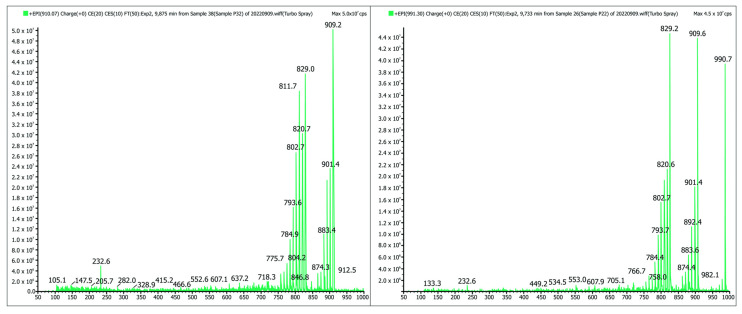
MS/MS spectrum of PRM prymnesin B (Cl + 1 hexose) and PRM B1 (Cl + 2 hexose) compounds in a sample from the Odra River. The analysis was performed using a QTRAP5500 spectrometer. The apparent loss of the Δ 81 fragment indicates the presence of hexose (source: own elaboration based on IOŚ-PIB official government report [16]).

Table 2 shows the ichthyotoxins of the prymnesin group detected in the samples from the Odra River, while Figure 4 shows the MS/MS spectrum of the PRM prymnesin B (Cl + 1 hexose) and PRM B1 (Cl + 2 hexose) compounds. The fragmentation spectra also indicated the presence of sugar residues in the molecule, a characteristic of prymnesins, as shown (in Figure 4) by the apparent loss of the Δ 81 fragment. The results refer to samples taken on 19 August 2022.

**Figure 5:**
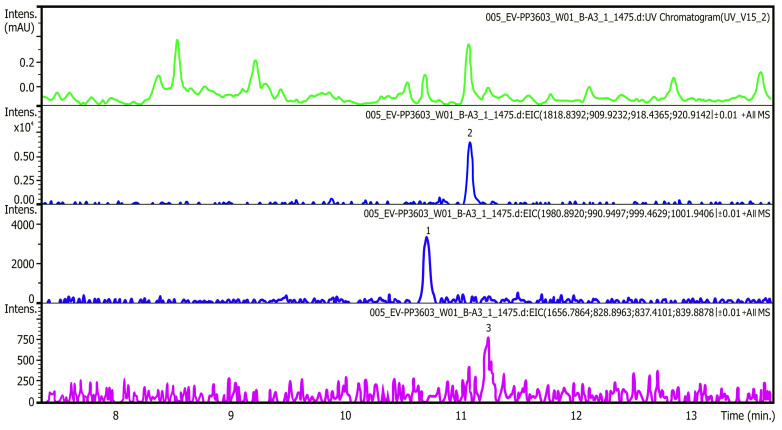
Chromatograms from the analysis of prymnesins content in water samples (source: based on [110]). The successive panels in the chromatogram show (from top): (1) UV at 280 nm; (2) chromatogram of extracted B-type prymnesin ions containing a chlorine unit and a hexose unit; (3) chromatogram of extracted B-type prymnesin ions containing a chlorine unit and two hexose units; and (4) chromatogram of extracted B-type prymnesin ions containing chlorine to which no sugar is bound (also called skeleton) (source: own elaboration based on IOŚ-PIB official government report [16,110]).

Water samples from the Odra River, its reservoirs, and the Gliwice Canal were analyzed for their toxin content. The analysis was performed according to the indirect method described by Svenssen et al. [115] and Medić et al. [116]. The results, presented in Table 3, show the relative amounts of each detected prymnesins in the samples and their estimated total value. The table also shows the abundance of *P. parvum*. Figure 5 shows example chromatograms from the analysis of prymnesin content in water samples. The top panel shows UV at 280 nm, and the following panels show chromatograms of extracted type B prymnesin ions containing one chlorine unit and one or two hexose units.

**Figure 6:**
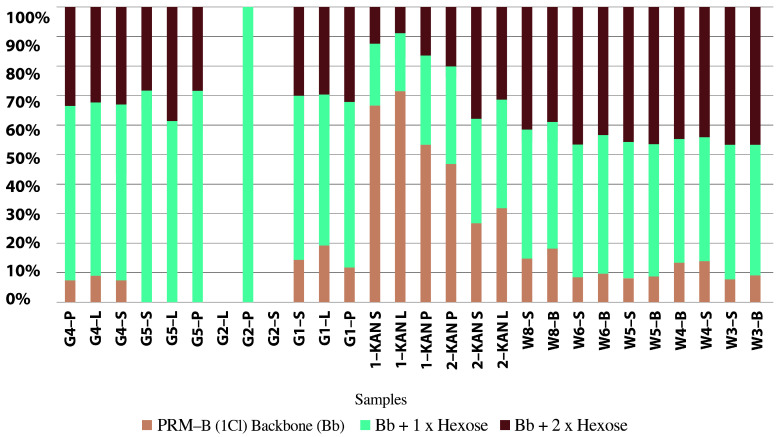
Distribution of group B prymnesins in selected water samples from the Odra River, reservoirs, and Gliwice Canal (source: own elaboration based on IOŚ-PIB official government report [16,110]).

**Figure 7:**
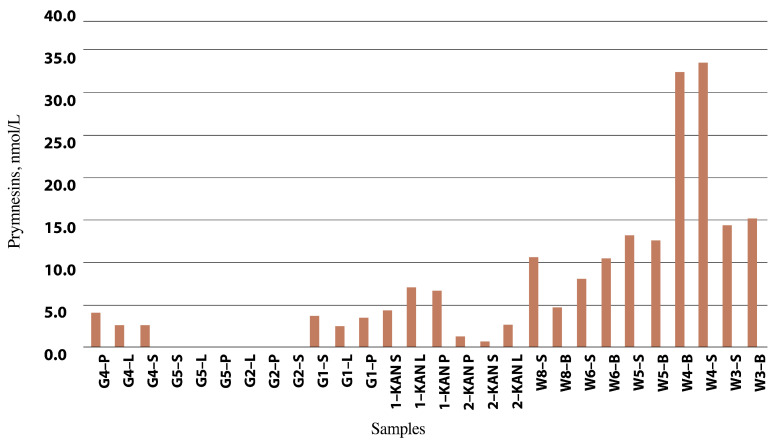
Estimated prymnesin content, expressed in nmol/L (C), in selected water samples from the Odra River, reservoirs, and Gliwice Canal (source: own elaboration based on IOŚ-PIB official government report [16,110]).

The detailed results of the chromatograms can be found in Table 3 and Figure 6 and Figure 7.

The highest estimated prymnesin content was 4.0 nmol/L in the Odra River water (Krosno Odrzańskie/Gostchorze), while concentrations ranging from 0.6 to 7.0 nmol/L were detected in the Gliwice Canal. The highest values were found in the Czernica Reservoir (33.5 nmol/L), followed by the Łacha Jelcz (15.0 nmol/L), Baikal (13.0 nmol/L) and Prężyce Reservoirs (10.5 nmol/L). Prymnesin PRM B, with an *m/z* value of 1818, contributed most to the total prymnesin [16]. Table 3 presents the results from the analysis of the estimated prymnesin content in group B. Peak area and estimated prymnesin content were measured using liquid chromatography high-resolution mass spectrometry (LC-HRMS) and liquid chromatography-fluorescence detection (LC-FLD), respectively [110].

Figure 6 shows the distribution of group B prymnesins in the selected water samples, and more specifically, the percentage of PRM-B (1 Cl) backbone (Bb), Bb + 1 × hexose, and Bb + 2 × hexose. In turn, Figure 7 shows the estimated prymnesin content, expressed in nmol/L, in the water samples from the Odra River, the reservoirs, and the Gliwice Canal. It is important to emphasize that this is only an indirect estimate, and some samples may have lower sensitivity due to the HPLC-FLD method.

**Figure 8:**
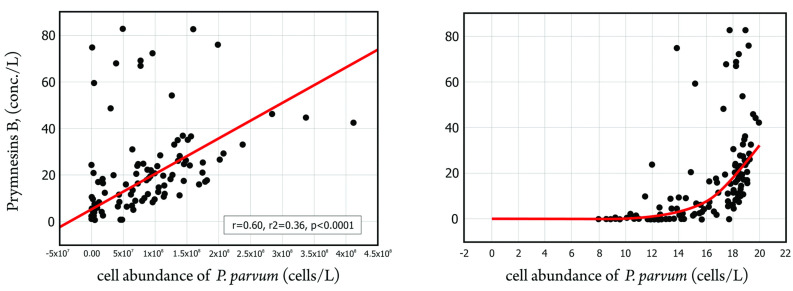
Relationship between cell abundance of *P. parvum* (cells/L) and the relative number of prymnesins (**left panel**) and the relative number of prymnesins in log-transformed values (**right panel**) (source: own elaboration based on IOŚ-PIB official government report [16,110]).

In addition, 203 water samples from the Odra River were analyzed to determine the relationship between the number of cells of *P. parvum* per liter of water and the relative total concentration of prymnesins in the water [16]. The number of cells was determined using microscopic analysis, while the concentration of prymnesins was determined using LC-MS/MS analysis [110]. Figure 8 shows the results of the statistical analysis. 

The left panel shows a moderate and significant relationship between the number of cells and the relative concentration of prymnesins (correlation coefficient of 0.597). The right panel shows the relationship in logarithmic form, with an exponential increase in relative prymnesin concentration observed with increasing cell numbers up to 100 thousand cells/L. Using the fragmentation spectra from the QTRAP5500 mass spectrometer and the accurate measurement from the Bruker HRMS QTOF mass spectrometer, the presence of prymnesin type B group ichthyotoxins was detected in the water samples. In addition, a statistically significant and moderately strong correlation was found between the number of *P. parvum* cells and semi-quantitative prymnesin concentration. All in all, the collected evidence points to a connection between the occurrence of prymnesines and fish kills in the middle reaches of the Odra River.

#### 3.2.2. The Polish Team’s Final Report

The Polish team’s final report on the situation in the Odra River, published in March 2023, highlights several important facts and issues regarding the condition of the river and the occurrence of algal blooms [117]. It addresses the link between algal blooms and pollution from municipal wastewater, and in particular, highlights the role of nitrogen and phosphorus compounds in promoting phytoplankton growth [117]. The presence of “golden algae”, particularly *P. parvum*, is of great concern.

An important finding is that municipal wastewater accounts for 60% of annual discharges in the Odra River basin, with the province of Lower Silesia accounting for the largest share [117]. Mining accounts for 11% of the discharged wastewater [117]. The report highlights the need for careful analysis of the characteristics of wastewater discharged into the river in order to effectively address the problem.

The study of “golden algae” in the Szczecin Lagoon and Oder Bay in 2022 complements the preliminary report on the area of transitional waters. The report refers to the development of scientific research on *P. parvum* and the success of sequencing its complete genome [117]. This breakthrough contributes significantly to the understanding of this organism and its potential impact on the ecosystem. As for the physicochemical conditions of the water, the report shows that the analyzed salinity indicators do not show major deviations from historical values. Variations in salinity are influenced by factors such as the discharge of saline groundwater and hydrological conditions. However, the report highlights that weather conditions, climate change, and low water levels can affect water quality, especially in the lower part of the river, which is influenced by the saline water of the Szczecin Lagoon [117]. 

The occurrence and frequency of *P. parvum* are influenced by various environmental conditions, including the salinity of the water and nutrient availability. The species thrives in waters with a conductivity range of 3.0-4.5 thousand µs/cm, but its occurrence is not solely dependent on salinity. Nutritional conditions, especially the ratio of nitrogen to total phosphorus, also play an important role [117].

Moreover, the report emphasizes the need for further scientific research to understand the bloom phenomenon and its possible causes, effects, and countermeasures. Due to the irregularities caused by the ecological disaster, comprehensive field experiments and planned monitoring were not possible. The scientific community must continue to study this issue to develop effective strategies to mitigate future algal blooms.

In addition, a study on the Szczecin Lagoon and Odra Bay reveals the presence of *P. parvum* strains that possess genes that enable the production of prymnesins [117]. Although the contribution of *P. parvum* to the total abundance and biomass of phytoplankton was relatively low, the risk of toxic blooms and their effects on fish and aquatic organisms, especially bivalves, remains of concern [117]. The report highlights that the dynamic environmental conditions of the lagoon, anthropogenic pressures, and changes in the hydrologic regime may contribute to the vulnerability of the area to negative phenomena, including toxic blooms.

The report also accentuates the negative impact of the ecological disaster on the ichthyofauna of the Odra River. The assessment of ecological potential using the index of biological integrity (IBI is a scientific tool used to identify and classify water pollution problems) shows a decline in the ecological potential of ichthyofauna in several sections of the river [117]. The loss of protected fish species, such as the spined loach (*Cobitis taenia*) and amur bitterling (*Rhodeus sericeus*), is attributed to the lack of refugia and regulation of the river that reduces habitat availability [117]. 

In summary, the final report on the situation in the Odra River presents important findings regarding the relationship between algal blooms and pollution from municipal wastewater, the occurrence and abundance of *P. parvum*, and the impact on water quality and aquatic organisms. It emphasizes the need for further research and effective mitigation strategies to address these issues. By providing a comprehensive overview of the current situation, the report serves as a valuable resource for policymakers, water management authorities, and environmental organizations. The findings underscore the urgency for implementing measures to reduce pollution inputs and develop sustainable practices to protect the Odra River ecosystem. Further research and collaboration among stakeholders are essential to ensure the long-term health and viability of the river and its surrounding communities.

### 3.3. German Report

The German researchers investigated the events on the German side of the Odra River [118]. In their results, they referred to the period between 1 and 22 August 2022. The report indicated an increase in conductivity (a measure of dissolved ions in water) as early as the first days of August (at the level of Frankfurt (Oder)) and two days later, downstream at the level of Hohenwutzen (measured on 3 August 2022). Maximum conductivities (>2000 μS/cm) were measured at the Frankfurt (Oder) monitoring site for the period between 7 and 14 August 2022 [118]. Significant increases in oxygen concentration, pH, and chlorophyll concentration, and decreases in nitrate concentration were observed, among others—an environment conducive to massive algal blooms. Large amounts of salt (mainly sodium chloride) were also detected, which previous studies (in the literature) have suggested is a suitable environment for a massive algal bloom [118]. A suitable remote sensing study, using appropriate high-resolution satellite imagery, provided accurate chlorophyll measurements that confirmed the algal bloom (and estimated the extent of the phenomenon). German researchers succeeded in demonstrating the high toxicity of total constituents in the waters of the Odra River using bioassays with daphnia (water fleas) [118]. Based on taxonomic and molecular biology methods, the alga *P. parvum* (normally found in highly saline waters) was identified in the phytoplankton samples, as in the Polish report. Cell counts (per liter of water) were consistent with values reported in the literature for mass fish kills caused by *P. parvum*. Toxins were identified in water samples from the Odra River (based on untargeted screening). The origin of the toxins was associated with golden alga, as it is known (from documented cases) to produce the harmful toxin prymnesin. On the other hand, it was not possible to quantify the concentration of toxins (and the criteria for their evaluation). For the same reason, it was also not possible to demonstrate conclusively that the concentration of the toxin was high enough to cause mass mortality of fish. Herbicides, specifically 2,4-dichlorophenoxyacetic acid (2,4-D) and 2,6-dichlorophenoxyacetic acid (2,6-D) and their technical byproducts, as well as 2,4,6-trichlorophenoxyacetic acid (2,4,6-T) were detected in the samples collected [118]. The concentrations of the detected herbicides were higher than those considered hazardous under existing assessment criteria, but ultimately their strong toxic effects on aquatic fauna were considered unlikely at the concentrations measured.

In summary, the report cited toxins secreted by *P. parvum* as the cause of acute toxicity to fish [118]. The German side performed a whole range of different chemical analyses, including extensive screening (about 1200 different substances), but no toxic substances other than the above-mentioned prymnesins were identified that could cause acute toxicity to fish. Observations were made at various monitoring sites including Frankfurt (Oder) and Hohenwutzen. Water samples from Hohenwutzen showed pronounced daphnia toxicity [118]. In addition, the effect of a heavy algal bloom on the occurrence of *P. parvum* and the prymnesin toxins derived from *P. parvum* was observed. The group of German scientists and experts analyzed numerous research hypotheses and finally concluded that the toxins produced by the massive proliferation of *P. parvum* (caused by high salt concentrations) were the most likely cause of the fish kill [118]. It is worth mentioning that the algal bloom of *P. parvum* in the Odra River occurred in the summer when the Polish environment has the right temperature and light conditions that favor it. However, it does not mean that this is the case everywhere in the world. In the United States, for example, *P. parvum* algal blooms and associated fish kills often occur during the cooler winter and spring months [119]. It is also worth noting that climate change may cause harmful algal blooms to occur more frequently, in more waters, and with greater intensity in the future, and warmer waters may favor more blooms as a result of climate change [82]. Therefore, it is possible that *P. parvum* algal blooms will occur at different times of the year in the future than they do today. Since *P. parvum* algal blooms usually occur in association with high salt concentrations in the water, the German researchers pointed out that it was not possible to precisely locate the original habitat of *P. parvum* algae in the Odra River or to determine the origin of the salt and other chemical compounds. They referred to previously collected data that showed that *P. parvum* is relatively rare (mostly in small numbers of cells) on the German side of the river (in saline coastal transition waters). This can be interpreted as a confirmation of the hypothesis for an anthropogenic cause of the ecological catastrophe in the Odra River.

It is important to note that both government reports (both the German [118] and the Polish) were published around the same time. As far as the mechanisms underlying the phenomenon are concerned, the two reports are indeed in agreement, but the German researchers strongly emphasize anthropogenic causes due to the above-average salinity of the Odra River, which led to the algal bloom. It is also worth noting that the experts point out that mining, especially the coal industry, is most responsible for the high salinity of rivers such as the Oder and Vistula [120]. When coal seams are mined, huge volumes of mine water are pumped out, containing significant amounts of chloride and sulfate salts—generally, greater amounts with deeper mining. This is because groundwater is constantly flowing into the mine workings and must be pumped out on an ongoing basis. This water is therefore pumped from the mine workings to the surface and directed into nearby natural or artificial drainage ditches. From there, it flows into streams and rivers. In Upper Silesia, Poland’s largest coal mining region, the pollutants contained in the mine water enter the Vistula and Oder rivers. This means that Poland’s two largest rivers are being salinated practically at their sources. A report published by the government states unequivocally that mines are responsible for discharging the largest number of chlorides and sulfides into the Odra River basin, accounting for up to 72% of the total content in their effluents [121]. Government-mandated monitoring of the Odra River shows that its salinity exceeds limits at every control point along virtually the entire length of the river. Coal companies certified by the State Water Holding Company “Polish Waters” (Wody Polskie) may continue to legally discharge high-salinity wastewater into Polish rivers [120]. There may also be causes other than those that can be linked to mine operations. For example, a German report found that perchlorate concentrations at the Hohenwutzen site doubled between 5 August 2022 and 10 August 2022, analogous to an increase in conductivity, possibly salt concentrations [118]. Perchlorate is used as an oxidizer in fireworks, rockets, ammunition, flares, and airbags, among other things [122]. In addition, perchlorate is found in elevated concentrations in some fertilizers and may be formed in small amounts during disinfection processes (which could indicate anthropogenic activity). However, perchlorate also forms in the atmosphere and is deposited as salt, so the increase in concentration is likely due to increased salinity.

### 3.4. The EU Report/Analysis of the Ecological Disaster in the Odra River in 2022

The publication “An EU analysis of the ecological disaster in the Oder River of 2022” [123] is a technical report by the Joint Research Centre (JRC), which is the European Commission’s science and knowledge service [123]. The report analyzes the ecological disaster that occurred in the Oder River during the summer of 2022 and warns of the threat to European rivers from invasive algal species [124]. The value added from the EU analysis of the ecological disaster in the Oder River in 2022, when compared to the Polish and German official reports, is that it provides evidence-based scientific support to the European Commission, building on the German and Polish analysis. In other words, the EU report analyzes the ecological disaster, draws lessons from it, and warns of the threat to European rivers from invasive algal species. Moreover, the EU report provides recommendations for the entire EU. One of the key improvements recommended by the report is in terms of water quality monitoring and data transparency that would have limited the damages from the ecological disaster [125]. The report also recommends the development of a comprehensive monitoring system for water quality and the establishment of a European network of water quality monitoring stations [123]. Additionally, the report suggests the need for better coordination and cooperation among the EU member states in terms of water management and the implementation of the EU Water Framework Directive [123]. Finally, the report calls for the development of a comprehensive strategy for the prevention and management of invasive species in European rivers [123].

The ecological disaster in the Oder River in 2022 has raised concerns about the health and sustainability of European rivers. The EU analysis of this disaster provides valuable insights into the causes, impacts, and actions needed to prevent similar incidents in the future. The report highlights that the investigations conducted by the Polish authorities after the disaster have been instrumental in understanding the causes and developing effective remediation strategies [123]. Comprehensive monitoring of water quality and ecological status provides valuable information for preventive measures. This underscores the importance of investigative monitoring in identifying causes and targeting remediation efforts. The report argues that EEA data reveal poor ecological conditions in many European rivers, including the Odra River, due to pollution and biodiversity decline [123,125]. Therefore, improved water quality monitoring and data transparency are essential to prevent similar disasters in the future [123,125]. Transparent transmission of data enables better decision-making, while comprehensive monitoring systems and a European network of stations facilitate proactive measures. The report mentions that historical data on the Odra River indicate a long history of pollution and degradation [123]. For this reason, it is important to prioritize long-term water quality monitoring and management to prevent further deterioration and restore ecological health [123]. The travel time of water in the Odra River also underscores the need for rapid notification in the event of pollution [123]. Immediate notification of downstream agencies and neighboring countries allows for quick action to mitigate impacts [123]. An important point made by the report is that modeling and transparent communication of water flow timing are critical for effective and coordinated water management. In addition, nutrient loading, particularly, nitrogen and phosphorus, contributes to algal blooms in the Odra River [123]. Controlling nutrient input and flow dynamics is critical to prevent blooms and restore ecological balance [123]. Reports from EU member states show that ecological conditions in European rivers are poor [123]. Strengthening coordination and cooperation in water management and implementing the EU Water Framework Directive are crucial [123,125,126,127]. The exchange of best practices, harmonization of standards, and development of a comprehensive monitoring system are necessary.

The increased salinity in the Odra River during the disaster may have contributed to algal growth and fish kills [123]. Monitoring salinity and improving coordination among Member States are essential for effective water management and implementation of the Directive [123].

The recommendations collected provide a roadmap for preventing future disasters [123,125]. Improving water quality monitoring, data transparency, and coordination among member states is of utmost importance. The development of effective remediation strategies and long-term monitoring will ensure the health and sustainability of European rivers.

In summary, the analysis of the ecological disaster in the Odra River highlights the importance of investigative monitoring, improved data transparency, and coordinated efforts among member states. Strengthening water quality monitoring, implementing the EU Water Framework Directive, and addressing nutrient pollution are critical to preventing similar disasters and restoring ecological balance. By taking immediate action based on the recommendations collected, policymakers and stakeholders can ensure the health and sustainability of Europe’s rivers and protect vital ecosystems for future generations.

## 4. Discussion

**Figure 9:**
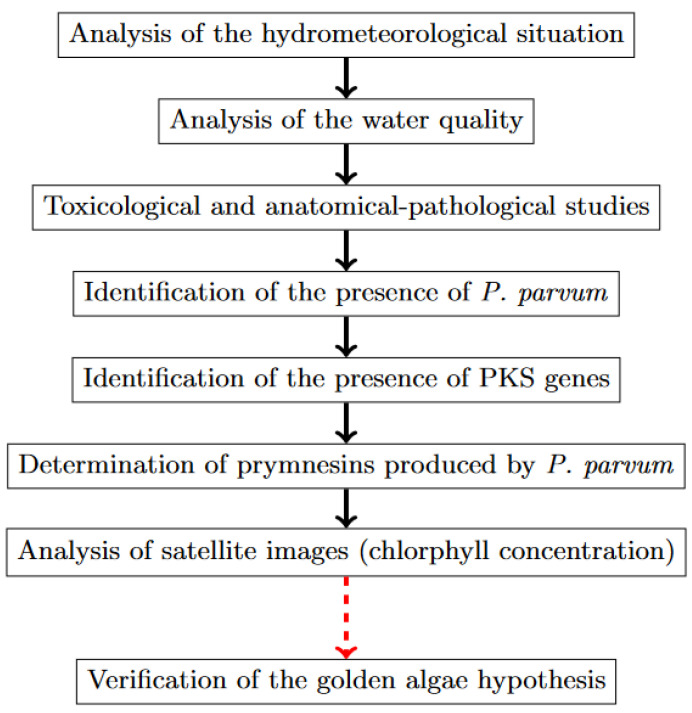
Diagram showing the “golden algae” hypothesis testing (source: own elaboration).

The topics covered in the government reports on the ecological disaster in the Odra River from July to September 2022 included (1) an analysis of the hydrometeorological situation in the period before the fish kill; (2) an analysis of the water quality of the Odra River in the period before the fish kill [16,117,118]; (3) toxicological and anatomical-pathological studies on the fish [16,118]; (4) the identification of the presence and bloom of *P. parvum* [16,110]; (5) the identification of the presence of PKS genes [16]; (6) a determination of prymnesins produced by *P. parvum* [16,110]; and (7) an analysis of satellite images [16,118,123]. All these analyses made it possible to formulate and verify the hypothesis about the relationship between fish mortality and the algal activity of *P. parvum* in the Odra River. The scope of analyses and studies conducted to test the golden algae hypothesis as a cause of mass fish kills in the Odra River in 2022 is summarized in Figure 9.

The occurrence of mass blooms of microalgae along the entire length of a large river such as the Odra River is a surprising case for scientists, but it was not the only one observed so far. Blooms of *P. parvum*, also known as golden algae, have already appeared in rivers, reservoirs, and marine waters around the world [52,119,128,129]. These blooms can be harmful to aquatic life and cause mass mortality of fish [119,128,129]. *P. parvum* is a unicellular algal species that lives in water and tolerates a wide range of salinities and temperatures [128,130]. Massive blooms of *P. parvum* currently occur in the United States [78], and blooms have also been observed in waters in Denmark, Finland, China, Israel, the United Kingdom, and Australia [104].

In Europe, *P. parvum* has been known since the 1920s. In 1990, a bloom of *P. parvum* and a fish kill were observed in the Netherlands in the Botshol Reserve in Utrecht, which consists of two shallow lakes, ditches, and reed belts created by peat cutting in the 17th century [103]. In Norway, a massive fish kill due to *P. parvum* blooms was observed in 1989, killing 750 tons of salmon and trout in cages and resulting in significant economic losses to the fish farming industry [131]. It is now known that the phenomenon of mass blooms of microalgae is favored by high temperatures and solar radiation, increased water salinity, and poor water flow [93,132,133]. *P. parvum*, like other algae, particularly proliferates in shallow, stagnant, or weakly flowing waters [132,133]. Importantly, a bloom of *P. parvum* is not always accompanied by the presence of ichthyotoxins, which are lethal to fish and other gill organisms. Algae (habitats of *P. parvum*) can produce prymnesins that are harmful to fish in certain situations. The production of prymnesins is favored by a sudden change in water parameters, which include an increase but also a decrease in salinity [134]. In other words, salinity is one of the factors affecting the activation and effectiveness of prymnesins [134]. However, there are no strictly defined limiting conditions in Europe or worldwide in which a bloom occurs. Wagstaff et al. [78] suggested that a reduction in salinity could lead to unfavorable conditions for golden algae-producing prymnesins. In addition, Flood and Burkholder [135] found that hemolytic activity increased in N-P-limited systems, suggesting that imbalanced nutrient systems may also increase prymnesin production. Thus, while a sudden change in water parameters may favor prymnesin production, it is not clear whether both an increase and decrease in salinity would have this effect. There are also no precise study patterns for the fish-killing prymnesins [16]. It appears that all the conditions described above occurred in the Odra River during the summer of 2022 [16,117,118,123]. Particular hydrological and meteorological conditions favored the proliferation of microalgae (the occurrence of mass blooms of microalgae) during this period, as confirmed in both government reports (both Polish and German) [16,117,118]. In short, the occurrence of mass blooms of microalgae in the Odra River was favored by hot weather and very high solar radiation, which exceeded normal values by 30% in July 2022 [16]. The water temperature in the Odra River averaged 27 °C [16]. The high temperatures and the absence of precipitation contributed to changes in water parameters, including an increase in salinity. There was no significant precipitation in June or July 2022, resulting in low water levels in the river. Flows were also very low, which means that the water in the river was almost constant [16,117,118,123]. This is very important because it means that the same number of substances discharged into the river at low water levels resulted in much higher concentrations. This phenomenon was confirmed with analyses of the conductivity, chloride concentration, and pH of the water during the period of the disaster [16,117,118,123].

As for the hydrological situation of the Odra River in the run-up to the disaster, hydrographs were well below the multi-year mean discharge (SSQ) and closer to the multi-year mean low flow (SNQ) almost during the whole analyzed period (from the beginning of July 2022 to 20 August 2022) [16,117]. Importantly, the observed downward trend in water levels weakened at some point [16,117]. Since 21 August, as a result of intense precipitation in the south of the country, there was an increase in water levels almost along the entire length of the Odra River, with a transition to the zone of medium water levels [117]. In the canalized section of the Odra River and at the mouth of the Szczecin Lagoon, the trend in the change was less obvious. After the passage of the storm surge, the stations reported some declines, while water levels generally remained above low water until late August [16]. Large fluctuations and increases in water levels caused by intense rainfall should be considered conducive to the development of ecological catastrophic blooms. The hydrological situation, i.e., the condition and corresponding flow rate, have a direct influence on the water quality of the river and thus on the physico-chemical and biological conditions [117]. In general, higher concentrations of certain substances in a river also require higher river water levels for a given pollutant load. A river with a lower water level is more susceptible to rapid changes in physicochemical conditions, i.e., the water warms faster, and the temperature rise is deeper. A higher water temperature is usually associated with lower oxygen levels. Regarding water quality, since 28 July 2022, the Central Research Laboratory of the General Inspectorate of Environmental Protection (GIOS) has been taking daily samples at additional sites on the river in addition to routine water quality monitoring under the State Environmental Monitoring Program, the number of which has fluctuated between 34 and 37 since mid-August [16,117]. In total, more than 20,000 physicochemical determinations have been made through the 3rd decade in September 2022 [16]. These include (1) thermal oxygen conditions (water temperature, dissolved oxygen, oxygen saturation, COD -Mn, total organic carbon, COD -Cr, total suspended solids); (2) salinity conditions (specific electrolytic conductivity, sulfate, chloride, sodium, potassium, hardness); (3) acidification conditions (pH); (4) biogenic conditions (Kjeldahl nitrogen, nitrate–nitrogen, nitrite–nitrogen, ammonia–nitrogen, total nitrogen, total phosphorus, phosphate–phosphorus); (5) specific pollutants (free cyanides, phenol index, petroleum); and (6) metals (chlorine, mercury, cadmium, lead, nickel) and elements (Li, Be, B, Al, Ti, V, Cr, Mn, Fe, Co, Ni, Cu, Zn, As, Se, Sr, Mo, Ag, Cd, Sn, Sb, Ba, Tl, Pb) [16,117].

It should be restated that the Odra River is a river in poor ecological condition, as it flows through industrial areas [120]. The salinity in some places, especially in the channels connected to the river in Silesia, is close to the values measured in the Baltic Sea [118]. However, it is worth noting that the condition of the water in terms of quality in 2022 did not differ from the monitoring data that had been characteristic of the river for years [16]. The scientists who conducted the analysis as part of the government teams did not find any additional factors that could have led to the mass death of fish and mussels in the Odra River [16,118].

The toxicological tests were conducted by the State Veterinary Inspectorate and included (1) 148 analyses of lead, cadmium, mercury, and arsenic and (2) 98 analyses of organochlorine pesticides and polychlorinated biphenyls (PCBs) [16]. In the toxicological tests conducted by the State Veterinary Institute, 109 samples were analyzed for toxic elements, pesticides, mold toxins, and other toxic compounds, and 6 samples were analyzed for persistent organic pollutants and radioactive contaminants [16]. Overall, the toxicological tests performed—for a total of more than 300 chemicals and trace elements—showed that the concentrations of the substances in the submitted samples did not deviate from the characteristic levels of environmental pollution in rivers in Poland [117]. According to the current state of toxicological knowledge, it could be ruled out that the above-mentioned compounds were the cause of the poisonings and fish kills.

Numerous clinical, anatomical–parasitological, bacteriological, mycological, and histopathological examinations were carried out as part of the governmental work [16]. During the anatomopathological examinations, including ichthyopathological examinations of the Institute of Inland Fisheries, 116 fish samples and 7 bivalves were examined [136]. Most of the examined animals were clinically healthy and showed no signs of disease. Ectoparasites and endoparasites were found in non-invasive amounts in some of the examined animals. Despite the absence of clinical changes, the histopathological picture of all examined animals indicated acute damage to the most perfused organs, i.e., the gills, spleen, and kidneys [136]. The disruption to hematopoietic processes and damage to the gills (an organ responsible not only for gas exchange but also for osmotic regulation and immunity) are usually associated with hemolytic toxins, which include prymnesin toxins secreted by *P. parvum* [99,104,130,134,137]. More specifically, this is indicated by necrotic lesions in the spleen involving both white and red pulp and activation of melanoma macrophage centers of the spleen and renal interstitium [136]. In contrast, no animal exhibited fatty degeneration or severe hydrops degeneration of hepatocytes, and no necrotic lesions normally associated with heavy metal poisoning in fish were observed [136]. Overall, toxicological tests for more than 300 substances, including heavy metals, ruled out pollutants as the cause of mass mortality in the Odra River [117]. Bacteria, viruses, and parasites were also ruled out as causes of the deaths. Between 2 August and 5 September 2022, a total of 334 samples were collected for laboratory testing, including [136] (1) 278 samples for toxicological studies and (2) 56 fish samples for anatomopathological and histopathological studies. As mentioned above, the tested fish showed acute damage to the most perfused organs, and the picture of the tested tissues indicated rapid death from a fast-acting substance [16]. This was not a prolonged exposure, but rapid environmental changes must have occurred to produce such a picture of the tissues. The disruption to hematopoietic processes and damage to the gills indicated a strong effect of the hemolytic toxins, which led to the hypothesis of a toxic effect of the prymnesins produced by *P. parvum* [117]. 

The presence of large amounts of ichthyotoxins in samples from the Odra River and Gliwice Canal was confirmed by researchers from the University of Gdańsk Laboratory (in September 2022) [16]. The results confirmed the analyses performed by the laboratory at the University of Vienna. In the course of further work, researchers from the University of Gdansk isolated DNA and RNA from prymnesins [16]. To date, the literature on the genetics of *P. parvum* and the genes encoding the enzymes necessary for the production of prymnesins is extremely sparse. In particular, no complete genome of this organism has been deposited in databases of nucleotide sequences, making genetic analysis much more difficult. There are a few papers in the literature that have attempted to describe the genes encoding the above enzymes, but the published analyses are very incomplete and also involve only single strains of *P. parvum* [115,134,138,139,140,141,142]. The genes encoding prymnesin-synthesizing enzymes, called PKS, are known to be modular [134]. They encode individual protein domains that have specific catalytic properties required for prymensin synthesis. To determine whether *P. parvum* genes might be present in the environmental samples obtained for the study, in which *P. parvum* had previously been found, a polymerase chain reaction (PCR) was performed using primers designed based on previous data from the literature [16,140]. The results showed amplification of DNA isolated from the collected samples in the PCR reaction with primers PKS1, PKS2, and PKS3 [16]. Signals were detectable (with varying intensity) in all tested samples, while no signal was present in the control sample, indicating that the tested biological material contained genes encoding enzymes that catalyze prymnesin production. RT-qPCR analyses showed the possible expression of genes encoding enzymes involved in the production of prymnesin in the tested samples [16]. They indicated that genes encoding enzymes/modules involved in the synthesis of prymnesin were present in the tested material. These modules were assembled into a single contig, demonstrating the functionality of this fragment in the genome of the *P. parvum* strain present in the tested samples. It is worth noting that Polish researchers are currently working on the development of a template that will allow accurate determination of these poorly studied ichthyotoxins, which may allow more precise determination of the conditions under which toxin release occurs [117]. This will also help determine the likelihood of *P. parvum* blooms in inland waters. As part of the government research, *P. parvum* was identified and quantified [16,110]. A total of 211 water samples were collected from different sections of the Odra River as well as from reservoirs, canals, and rivers closely connected to the Odra River and analyzed for phytoplankton. Sampling took place between 12 August and 8 September 2022. Control water samples were also collected for analysis on consecutive days through September 21 to monitor the prevailing status (at that time). The presence of *P. parvum* was detected in 165 (78% of the total samples) of the 211 water samples analyzed, and its abundance was calculated [110]. According to the literature, fish kills were most frequently detected when the abundance exceeded 50–100 million cells/L [143]. In the samples studied, more than 50 million cells of *P. parvum* were found in 1 L of water in about 35% of the cases, while more than 100 million cells/L were detected in 22% of the total samples studied [110].

In the study, prymnesin (Prm) produced by *P. parvum* was determined [110]. The analyzed material was subjected to extraction and LC-MS/MS analysis (231 filter tubes containing material collected between 17 August and 7 September 2022). The analyses showed that at least three prymnesium B toxins were present in the samples from the *P. parvum* bloom area [110]. These compounds were recorded as doubly charged ions [M+2H]^+^ and pseudomolecular ions [M+H]^+^ and as sodium adducts [M+Na+H]^+^ (the apparent loss of Δ 81 fragments was indicative of the presence of hexose in the molecule). These ions are characteristic of group B prymnesins. The results were presented in terms of the relative amount of prymnesins in the tested material, expressed as the ratio of the chromatographic peak in the compound to the volume of the sample that was percolated. The presence of prymnesin group B ichthyotoxins was detected using fragmentation spectrum analysis and accurate mass measurement (HRMS/MS—high-resolution mass spectrometry). A statistically significant and moderately strong correlation was found between the number of *P. parvum* cells and the semi-quantified prymnesin concentration at the studied sites, as well as a correlation between the presence of prymnesin in water and fish mortality in the Middle Odra River [110].

Studies conducted worldwide on golden algal blooms show that the possibility of a bloom occurring, as well as the intensity of toxin production by these organisms and ultimately the intensity of the toxic effects of these compounds on aquatic organisms, are determined by a number of factors. According to one of these studies, the risk of a golden algal bloom increases at conductivities above 1500 µS/cm [144,145]. Moreover, the toxicity of *P. parvum* appears to be increased at a pH above 7.0 and under nutrient-poor conditions [146]. The physical and chemical properties of the water in which *P. parvum* was found (in China) are listed in Table 4. 

**Figure 10:**
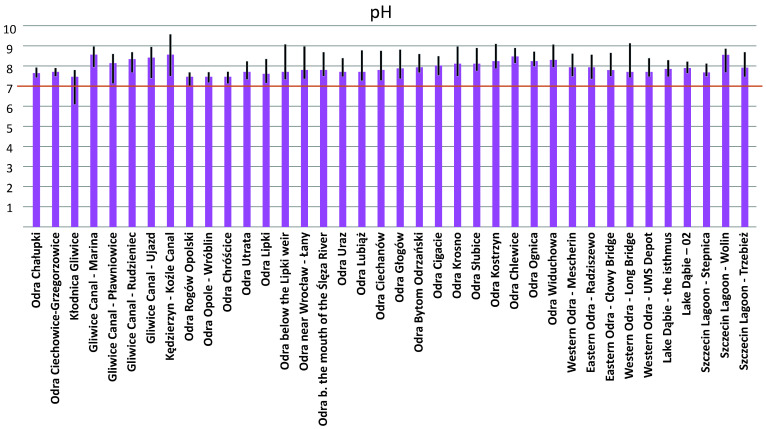
Changes in water pH at General Inspectorate of Environmental Protection (GIOS) monitoring and control points between 28 July and 20 September 2022 (source: own elaboration). Subsequent bars indicate measured data (unclassified indicator). The toxicity of *P. parvum* appears to be increased at a pH above 7.0 [16,95,146], as indicated with the red line.

**Figure 11:**
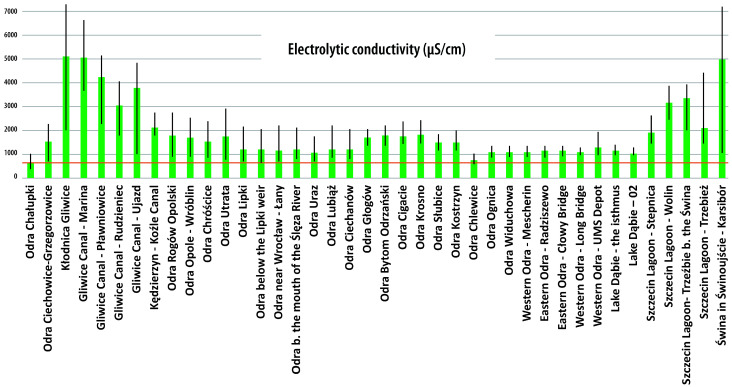
Values for electrolytic conductivity at measuring and control points of the General Inspectorate of Environmental Protection (GIOŚ) between 28 July and 20 September 2022 (source: own elaboration). NOTE: The red line shows the limit of good status for large lowland rivers (850 µS/cm).

Figure 10 and Figure 11 confirm that both the pH and electrolytic conductivity that prevailed in the Odra River during the summer of 2022 favored the bloom of *P. parvum*.

In Figure 11, the green boxes reflect the average values, while the whiskers (black lines) extend to the minmum and maximum values. Using Kłodnica Gliwice as an example, the average value for the entire period studied was 5000 μS/cm, the minimum value was ca. 2000 μS/cm, and the maximum value exceeded 7000 μS/cm [16]. The question arises: why is electrical conductivity even important when verifying the golden algae hypothesis? It is because there is scientific evidence that electrical conductivity (it can be viewed as a proxy for salinity: dissolved ions increase salinity as well as conductivity; therefore, these two measures are related) has increased and such increases are typically associated with golden algae blooms [144,147]. However, there is no conclusive evidence that huge fluctuations in the values of electrolytic conductivity favor the golden algae incidents. The exact environmental conditions favoring toxic algal blooms are not clear, and even though factors such as water temperature and salinity are somewhat helpful in predicting the occurrence of golden algae blooms, other factors such as nutrient availability and light intensity may also play a role [119].

**Figure 12:**
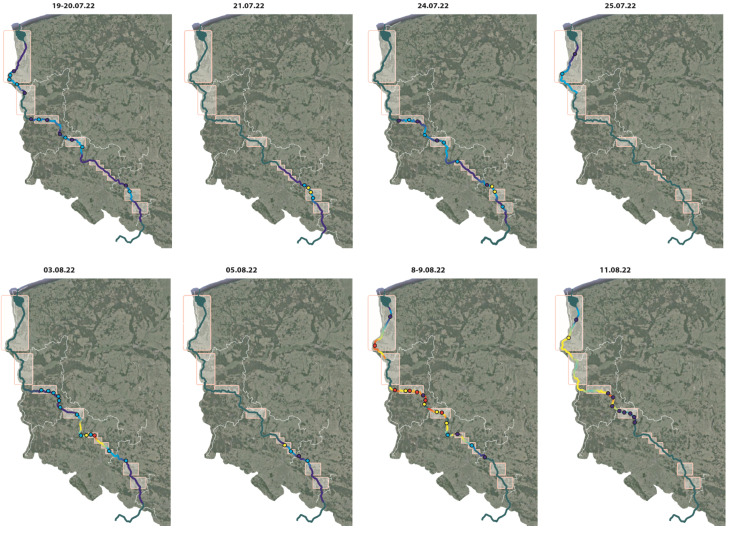

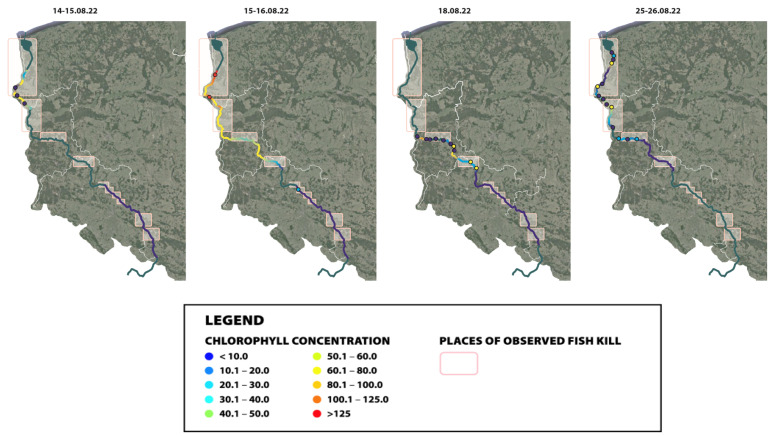
Chlorophyll concentration in the Odra River from 19 July to 26 August 2022 (source: own elaboration based on the IOŚ-PIB official report).

According to the literature, toxicity might be influenced by the developmental phase of the *P. parvum* population (small in the first period, with intense population growth, increasing once the population stabilization phase is reached) [134]. The increase in toxicity may be triggered by changes in the environment caused by the algal bloom itself—an increase in the pH of the water, a depletion in nutrients including nitrogen, and a sharp change in the N:P ratio [134]. The hypothesis of a link between fish kills and *P. parvum* algal activity in the Odra River can be additionally tested thanks to the use and analysis of the corresponding satellite images from the Sentinel-2 satellite [16,123]. Figure 12 shows chlorophyll concentrations along the entire length of the river for selected dates (corresponding to the development of the bloom from 19 July to 26 August 2022).

More specifically, Figure 12 shows that in the period from 19 July to 26 August 2022, there was a significant gradual increase in chlorophyll concentration in the section from the Groszkowice lock to the confluence of the Nysa Kłodzka with the Odra River, as well as near the confluence of the Gliwice Canal with the Odra River [16]. In the first days of August 2022, high chlorophyll concentrations (above 125 mg/m^3^) were again measured in the middle part of the Odra River. In the second week of August 2022, high chlorophyll levels were measured in the lower part of the river [16]. Around 20 August 2022, the situation in the upper part of the Odra River started to normalize.

**Figure 13:**
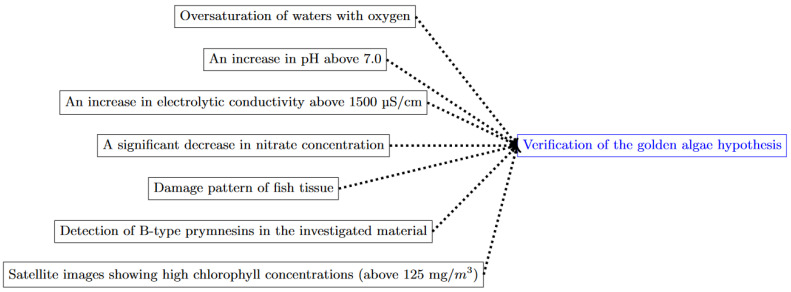
Summary showing the “golden algae” hypothesis testing (source: own elaboration).

In conclusion, the intense golden algal bloom in the waters of the Odra River was multifactorial (see Figure 13 for a summary showing the testing of the “golden algae” hypothesis). At the end of July and the beginning of August, favorable conditions for the development of *P. parvum* and the occurrence of toxicity prevailed in the Odra River waters, such as significantly increased conductivity, high chloride, and sulfate content, along with increased water temperature and significant fluctuations in water parameters over time. The hydromorphology of the Odra River, especially the presence of numerous reservoirs, as well as the slowing down of the river flow in front of weirs and channels, i.e., places that favor the bloom, are also not without significance. 

The accumulated scientific evidence suggests that the disaster in the Odra River was due to a massive bloom of the microalga *P. parvum*, which produces fish-killing toxins [16,117,118,123]. The phenomenon was facilitated by certain hydrological and meteorological conditions that contributed to changes in water parameters, including increased conductivity, high chloride, and sulfate levels, increased water temperature, and significant fluctuations in water parameters over time [117,118]. Additional support for the evidence in this study is also provided by corresponding satellite imagery [16,123]. The hypothesis for the effect of ichthyotoxins is also supported by the fact that only aquatic organisms with gills died en masse in the Odra River [117]. The histopathological picture of the examined fish showed acute damage to the most perfused organs (gills, spleen, kidneys) [16]. The disruption to hematopoietic processes and damage to the gills are most likely related to the effects of hemolytic toxins.

Mass blooms of golden algae in the waters of the Odra River and other rivers and reservoirs may recur in subsequent years, which has happened in other countries around the world [117,123]. Tests of water samples from the Vistula River showed no evidence of *P. parvum*. As mentioned earlier, the EU JRC report shows that ecological conditions in European rivers are poor [123]. It provides a comprehensive overview of the causes, impacts, and necessary actions to prevent similar incidents in the future [123,125]. In addition, there are methods to suppress golden algal blooms and reduce their toxicity using selected chemicals, minerals, nutrient manipulations, and water flushing that are feasible in smaller water bodies [148,149,150,151,152]. There is a number of studies discussing various methods for controlling and treating harmful algal blooms, including the use of flocculating agents, clay slurries, and nutrient manipulations [119,149,151]. They also discuss the effectiveness of these methods in reducing the toxicity of golden algal blooms in smaller water bodies. However, there is no evidence in the literature for the feasibility of suppressing algal blooms in larger reservoirs and rivers of similar size to the Odra River. After analyzing the situation in the Odra River, it is recommended that solutions be introduced to reduce the risk of a repeat disaster of similar magnitude to the one that occurred in 2022 [153]. Recommendations include [16,117,123]: (1) the establishment of an integrated system for continuous measurement of water quality for selected parameters with access to online data; (2) continued inspection of facilities discharging polluted water into the Odra River and its tributaries; (3) review and verification of existing permits for discharging wastewater into water bodies in the Odra River Basin; (4) an improvement of information flow; and (5) the gradual restoration of populations of fish and other groups of organisms. A system for continuous measurement of water quality based on selected parameters with access to online data should include monitoring of parameters related to blooms and monitoring of *P. parvum* itself. The intelligent watershed management system should use both ground-based and satellite data. Monitoring should initially cover water bodies where water parameters favorable to blooms are recorded. In addition, adequate organizational and financial conditions must be established for the ‘continuous operation of the system’. Control of the points discharging polluted water into the Odra River and its tributaries involves, among other things, identifying the points that are most responsible for the state of water quality of the Odra River. In this context, the existing permits for discharging wastewater into the waters of the Odra River Basin should be reviewed and verified in more detail. In addition, the parameters and intensity of discharges should be made dependent on current water test results, including the obligation to temporarily stop or reduce discharges in an emergency situation. There is also a need to improve information flow, establish an early warning and response system, and streamline crisis management procedures. It is also necessary to ensure that populations of fish and other groups of organisms affected by the disaster gradually recover. To achieve this goal, hydrobiologists, ichthyologists, and geneticists must work together with the participation of scientists from Poland and abroad.

## 5. Conclusions

This article provides insights into the toxic bloom of *P. parvum*, which was indicated as the main cause of the ecological disaster in the Odra River from July to September 2022. In particular, a series of studies and analyses is presented in the framework of testing the golden algae hypothesis. All these analyses made it possible to formulate and test the hypothesis on the relationship between fish mortality and the algal activity of *P. parvum* in the Odra River. Moreover, this article systematizes knowledge about the different perspectives used to verify the identification of the harmful toxins prymnesins (secreted by *P. parvum* habitats). 

In summary, the catastrophe was triggered by significantly increased conductivity, increased chloride and sulfate content, increased water temperature, high solar radiation, significant variations in water parameters over time, and a significant degree of hydromorphological alteration in the Odra River, which is a largely regulated river—the presence of numerous reservoirs, as well as the slowing of the river upstream of weirs, channels, and thus in places that favor blooms. Noteworthy is the significant increase in conductivity during the periods of fish mortality (this is evident in the reports from the Polish and German sides). The environmental disaster in the Odra River in 2022 (and the damage caused by it) poses many new challenges for politicians, officials, and scientists in terms of water management (and shaping future policy). First of all, it should be emphasized that this environmental disaster had multiple causes (multi-causal relationships), which is what the results presented in the reports (both in Poland and Germany) indicate. It is worthwhile to develop and implement appropriate preventive measures, including consideration of adequate monitoring that can be carried out continuously (and that was missing—one of the conclusions on the causes of the disaster). It is worth taking appropriate measures to prevent similar disasters in the future, not only in the Odra River but also in other Polish rivers and other water bodies. The need for strict control of discharges (including a thorough review of previously issued permits for such discharges), comprehensive monitoring using innovative technologies, and methods such as remote sensing, is also recommended as a preventive measure to avoid similar ecological disasters in the future. As part of such comprehensive monitoring and control of discharges, appropriate early warning plans should be developed and implemented. Scientific research should be given high priority, particularly to accurately determine the causes of the spread of toxic algae (such as *P. parvum*) in the aquatic environment and to better understand how to improve the resilience of aquatic ecosystems (particularly in the context of regulation and use, pollution loads, and permitted water withdrawals and discharges of various substances). As mentioned above, the lack of continuous measurements of water parameters in the Odra River is cited as one of the causes of this disaster (none of the Polish monitoring stations conducted such continuous monitoring). It is important to point out that from the information in the report of the Polish side, it appears that the disaster (assuming that it consisted of several causes) could have been avoided with high probability.

This article states that the increase in conductivity was one of the causes of the disaster. In this context, it should be noted that the disaster could have been avoided if the Polish government had fulfilled its own commitments made several years earlier. This refers to the Government Regulation on the “Odra River Basin Management Plan” published at the end of 2016 (Journal of Laws 2016, item 1967), according to which the quality of the structure and functioning of the surface water ecosystem (the so-called good ecological potential) in some of the more sensitive sections of the Odra River (including the Gliwice Canal and the Eastern Canal) should be ensured by the end of 2021. Unfortunately, these obligations were not fulfilled [16], which may have been one of the reasons for the ecological disaster in July–August 2022. It is also worth mentioning that since 2017, a new Water Act has been in force in Poland [108], in which the fulfillment of the above-mentioned obligations has been ceded to the State Water Holding Company “Polish Waters” (Wody Polskie), and this institution now exercises (by law) the ownership rights for flowing waters in Poland (this follows from the Water Act and, in particular, from Article 231).

The conclusion is that the ecological disaster in the Odra River is the result of a synergy of several factors. As for the fish kill, very often a certain chain of events is responsible for the fish kill phenomenon. For example, the fish kill in Lake Peipsi, Estonia, in the summer of 2002 was attributed to the synergistic effect of a blue–green algae bloom, high temperature, and low water level [84]. The fish kill in the Odra River could be an opportunity to review the warning plans for all Polish rivers and adjust them accordingly.

The study of *P. parvum* and its toxic blooms in the Odra River (in Poland and Germany) has significant implications at both local and global scales. At the local level, the Odra River is an important source of freshwater for communities, agriculture, and industry in Poland and other neighboring countries (to a lesser extent). Toxic blooms of *P. parvum* have been associated with fish kills, reduced water quality, and other ecological impacts that can have serious economic and social consequences for these communities. Therefore, understanding the causes and effects of these blooms is critical for effective management and mitigation strategies. On a global scale, *P. parvum* is one of many species of harmful algae that are becoming increasingly common in freshwater and marine environments around the world. These algal blooms are often triggered by nutrient pollution, climate change, and other anthropogenic factors that are expected to increase in the coming decades. The study of *P. parvum* and its interactions with the environment can provide important insights into the mechanisms underlying harmful algal blooms and their impacts on ecosystems and human health.

In addition, the study of *P. parvum* in the Odra River has far-reaching implications for freshwater ecology and conservation. As the world’s population continues to grow and the demand for freshwater increases, it becomes increasingly important to protect and restore freshwater ecosystems such as the Odra River. By studying the causes and consequences of *P. parvum* blooms, researchers can develop strategies to prevent and mitigate the effects of harmful algal blooms in other freshwater ecosystems around the world. In summary, the study of *P. parvum* in the Odra River and Poland is important at both local and global scales because it provides insight into the mechanisms underlying harmful algal blooms, the impacts on freshwater ecosystems, and the need for effective management and conservation strategies. By continuing to study *P. parvum* and other harmful algae, researchers can help protect and restore freshwater ecosystems for the benefit of humans and the environment.

This study also addresses the Polish team’s final report, which serves as a comprehensive analysis of the disaster, presenting key findings and insights. It highlights the significance of the research conducted and its implications for the industry. The report’s added value lies in its ability to provide actionable recommendations based on rigorous data analysis, which can assist decision-makers in making informed choices. Overall, the report’s contribution is instrumental in driving strategic initiatives, fostering innovation, and enhancing operational efficiency within the organization.

Last but not least, the EU JRC analysis of the ecological disaster in the Odra River provides a comprehensive overview of the causes, impacts, and necessary actions to prevent similar incidents in the future. Using extensive investigative monitoring, the report sheds light on the importance of understanding the causes of ecological disasters and developing effective remediation strategies. Furthermore, the analysis highlights the need for improved water quality monitoring, data transparency, and coordinated efforts among all EU member states. This EU JRC report serves as a valuable resource for policymakers and stakeholders, offering a roadmap to prevent ecological disasters and ensure the ecological health and sustainability of European rivers.

## Figures and Tables

**Table 1:** *P. parvum* cases in different countries.

Case Study Description	Country/Authors
In 2009, a devastating *P. parvum* bloom occurred at Barramundi Farm in Australia, resulting in the loss of all fish in the ponds. In response, a number of measures were taken to prevent similar incidents in the future [94]. To this end, an experimental manipulation of nutrients and pH was conducted in one of the ponds. The experimental pond was treated with Phoslock™ clay modified with lanthanum cations that irreversibly bind dissolved phosphorus in the water, and the pH was lowered to below 7.7 by adding molasses, which stimulates microbial growth. Despite these preventive measures, a bloom of *P. parvum* occurred in the culture ponds at water temperatures of 24 to 32 °C and salinities of 10 to 36 ppt, resulting in the death of all fish.	Australia: Seger et al. [94]
According to Guo et al. [95], citing numerous Chinese authors, there are three prymnesium species in China: *P. parvum*, P. saltans, and P. papillarum. In 1963, a phytoplankton bloom caused by *P. parvum* resulted in the loss of 100,000 carp fry in a fish farm at the Liaoning Province Fishery College in Dalian. Since then, this alga has been found every year in different parts of China, such as Tianjin, the Ningxia Autonomous Region, Inner Mongolia, Shanxi and Zhejiang provinces, and the Tibet Autonomous Region. *P. parvum* thrives in coastal brackish water in Dalian, Tianjin, and Zhejiang and in saline, sulfate-bearing inland water in Ningxia, Inner Mongolia, and Shanxi. P. saltans, on the other hand, has been isolated from the coasts of Guangdong, Tianjin, and Ningxia and occurs in similar habitats and locations as Ningxia. P. papillarum has been isolated from the coast of Shandong Province. Chen and Zeng [96] described a new species in China that caused rotifers and copepods that fed on it to die within 2 to 4 days [95].	China: Guo et al. [95]; Chen and Zeng [96]
In 2007, an increase in the occurrence of the alternative algal species *Prymnesium polylepis* was detected during a marine monitoring program [97]. This species is considered the second most important ichthyotoxic haptophyte after *P. parvum*. The peak of the extensive bloom occurred between March and May 2008 and was widespread in the southern, central, and northwestern Baltic Sea, with cell concentrations reaching up to 5 million cells per liter. At some sites, P. polylepis accounted for 30 to 90% of the total phytoplankton volume. However, in the northeastern Baltic Sea and the Gulf of Finland, P. polylepis was detected in low numbers. Larsson et al. [97] studied the effects of this extensive bloom on duck-billed birds, i.e., eiders (*Somateria mollissima*), in the Baltic Sea. They observed a sharp decline in breeding eiders at 28 colonies in the southern and central Baltic Sea between 2007 and 2008. The authors argue that the intense spring bloom of *P. polylepis* affected the eiders’ main food source, i.e., mussels, at feeding sites in both toxic and non-toxic ways, which affected the body condition of adult female eiders and their breeding readiness.	Denmark: Larsson et al. [97]
In 1990, Lindholm and Virtanen reported a bloom of *P. parvum* in the Strait of Dragsfjaerd in Finland that resulted in a fish kill. The concentration of *P. parvum* reached a peak of 50,000 cells/mL. Chemical analyses conducted during the disappearance of the bloom showed a decrease in total phosphorus and total nitrogen levels and a decrease in chlorophyll a levels. Levels were about 50% lower in areas outside the strait where *P. parvum* was present in lower numbers. Live cells of *P. parvum* showed autofluorescence that could be of diagnostic value. Seven years later, in 1997, a brackish water lake in SW Finland, Vargsundet, was contaminated with algal toxins, resulting in high fish mortality. During this event, dense populations of *Prymnesium* sp. and the toxin-producing cyanobacterium *Planktothrix agardhii* were observed, mainly in separate layers [98].	Finland: Lindholm and Virtanen [98]
Great Britain is historically significant because it is the country where a case of *P. parvum* was first documented (according to Carter’s 1937 publication). More specifically, *P. parvum* habitats were found in a brackish pond near Bembridge on the Isle of Wight. In the 1960s, reports of the species’ occurrence surfaced in Hickling Broad, part of the Norfolk Broads. The Norfolk Broads, which consist of shallow brackish water created by centuries of peat extraction, are now used for recreational purposes and generate an estimated GBP 550 million in annual revenue for the local economy. According to Wagstaff et al. [99], *P. parvum* blooms have likely been present in the Norfolk Broads since the early 20th century. The frequency of blooms in the region is high, as evidenced by the toxic *P. parvum* bloom in Hickling Broad in 2015 that resulted in the death of thousands of fish. To contain the damage, 600,000 fish were manually relocated and rescued [100].	Great Britain: Wagstaff et al. [99]; Wagstaff et al. [100];
According to Shilo and Shilo [101], *P. parvum* first appeared in Israel in 1947 and then spread rapidly in brackish water areas, causing major problems for fish farming. The authors argue that the control of *P. parvum* can be achieved either by destroying the organism or its toxin. In 1947, Reich and Aschner discovered that ammonium sulfate had a destructive effect on *P. parvum* even at low concentrations and was not harmful to other life forms. Gordon and Colorni [102] reported a bloom of *P. parvum* in the Arava Valley in southern Israel that resulted in gradual death of ornamental fish, Poecilia sp. and koi. The toxic effect was due to changes in the water system, including higher temperatures, a tripling in salinity, and eutrophication, which favored the growth of *P*. *parvum*. Treatment with 10 ppm ammonium sulfate stopped the fish kill.	Israel: Shilo and Shilo [101]; Gordon and Colorni [102]
In 1990, a significant algal bloom and fish kill were observed in the Botshol Reserve in Utrecht, the Netherlands, which consists of two shallow lakes, ditches, and reed beds. The reserve was originally a system of clear lakes, but due to eutrophication, the water quality has deteriorated since the 1960s. Efforts were made to restore the reserve by reducing external phosphorus inputs. This resulted in a significant reduction in phosphorus levels in the lake water, an improvement in the light climate, and a change in the composition of phyto- and zooplankton. However, these measures also led to the appearance of *P. parvum* blooms [103].	Netherlands: Rip et al. [103]
In Norway, the occurrence of *P. parvum* has been documented along the entire west coast, from Oslofjord in the south to Spitsbergen in the north. However, blooms of the species have only been observed in the Sandsfjord fjord system, which is characterized by a permanent brackish water layer at a depth of 2–5 m and a summer salinity of 4–7 psu. The first recorded bloom of *P*. *parvum* in Sandsfjord occurred in late July 1989 and had severe consequences for local fish farms. According to Johnsen et al. [104], the bloom resulted in the death of 750 tons of Atlantic salmon and rainbow trout. In subsequent years, blooms of *P. parvum* occurred repeatedly in July and August, resulting in losses to salmon farms. Due to the continued bloom, fish farmers finally decided to leave the area in 1995, which marked the end of the bloom. However, in 2005, an attempt to return to the region to farm fish resulted in another *P. parvum* bloom in 2007 and the loss of 135 tons of salmon.	Norway: Johnsen et al. [104]
The spread of the harmful *P. parvum* algae has caused major fish kills and financial losses in Texas and throughout the United States. According to Texas Parks and Wildlife agencies, fish kills in the upper Brazos River in 1981–1982 and in Red Bluff Reservoir in 1985 were likely caused by *P. parvum*, although this has not been confirmed. In 1985, *P. parvum* was confirmed as the cause of a 660 km fish kill in the Pecos River that resulted in the death of an estimated 110,000 fish. Between 1985 and 2000, *P. parvum* blooms caused fish kills in the Brazos, Colorado, and Rio Grande watersheds, killing an estimated 2.6 million fish. In the early 2000s, the rapid spread of *P. parvum* led to blooms in 15 other U.S. states, including Alabama, Arizona, and California. Now, the alga is present in all southern regions of the country and in some northern regions [77]. In 2001, a bloom of *P. parvum* caused significant damage to Texas fisheries. A winter bloom caused a massive fish kill in Lake Possum Kingdom and other reservoirs, as well as the death of over 5 million striped and hybrid perch fry in the Wichita River. In 2003, *P. parvum* invaded the Canadian River watershed and caused a minor fish kill. In subsequent years, more than 30 watersheds were affected by the alga. It is estimated that the *P. parvum* bloom caused the mortality of more than thirty-four million fish and caused tens of millions of dollars in financial losses [77]. Cases of *P. parvum* have been reported throughout the decade 2011–2020 in several regions of the United States [105,106], including Brady Creek Reservoir (2012), Colorado City Reservoir (2016), Concho River (2019), Baylor Creek Reservoir (2009, 2012), Buffalo Springs Reservoir (2011–2012, 2014–2016), Balmorhea Revervoir (2010), Diversion Reservoir—Lake Diversion (2010–2016), and in Southern California, in Lake Forest and Lake Elsinore (2014). Detailed information can be found in the Nonindigenous Aquatic Species Database [105].	United States: Roelke et al. [77]; Nonindigenous Aquatic Species Database [105]; Caron [106]

**Table 2:** Ichthyotoxins of the prymnesin group detected in the studied material from the Odra River. The table shows the values measured with a high-resolution mass spectrometer (source: own elaboration based on IOŚ-PIB official government report [16,110]).

Compound Symbol. Sum Formula.	Values of *m/z* for the Found Ions Error Relative to the Theoretical Value (*m/z*)		
	[M+H]^+^	[M+2H]^2+^	[M+Na+H]^2+^
Prymnesins PRM B1 (1 × Cl + 2 hexose); C_97_H_142_ClNO_39_	19,808,797 *0.0124*	9,909,502 *0.005*	10,019,370 *−0.0036*
Prymnesins PRM B (1 × Cl + 1 hexose); C_91_H_132_ClNO_34_	18,188,300 *−0.0092*	9,099,144 *−0.0088*	9,209,142 *0.0092*
Prymnesins PRM B (1 × Cl) C_85_H_122_ClNO_29_	16,567,775 *−0.0089*	8,288,902 *−0.0066*	8,398,799 *−0.0079*

**Table 3:** The results from the analysis of the estimated prymnesin content of group B (peak area of the three prymnesin variants in group B and their sums, estimated prymnesin content, % of prymnesin variants, and the ratio Bb/Hex and 2Hex/Hex) in selected water samples (source: own elaboration based on IOŚ-PIB official government report [16,110]).

Sample Name	Peak Area ^(1)^				Estimation SUM of Prymnesins ^(2)^			Percentage			**Ratio**	
	PRM-B (1 Cl) Backbone (Bb)	Bb + 1 × Hexose	Bb + 2 × Hexose	SUM				PRM-B (1 Cl) Backbone (Bb)	Bb + 1 × Hexose	Bb + 2 × Hexose	Bb/Hex	2Hex/Hex
	EIC (1656.7864; 828.8968; 837.4101; 839.8878) ± 0.01 +All MS	EIC (1818.8392; 909.9232; 918.4365; 920.9142) ± 0.01 +All MS	EIC (1980.8920; 990.9497; 999.4629; 1001.9406) ± 0.01 +All MS		Nmol in the Dried Down Sample	Sample Volume ^(3)^	Concentration in the Environment [nmol/L] ^(3)^	EIC (1656.7864; 828.8968; 837.4101; 839.8878) ± 0.01 +All MS	EIC (1818.8392; 909.9232; 918.4365; 920.9142) ± 0.01 +All MS	EIC (1980.8920; 990.9497; 999.4629; 1001.9406) ± 0.01 +All MS		
G4-P	2825	20,946	11,816	35,587	1.2	0.3	4.0	8	59	33	0.13	0.56
G4-L	2325	14,894	8093	25,311	0.8	0.3	2.6	9	59	32	0.16	0.54
G4-S	2844	21,569	11,817	36,230	0.8	0.3	2.6	8	60	33	0.13	0.55
G5-S	0	4362	1699	6062	n.m.	0.3	n.m.	0	72	28	0.00	0.39
G5-L	0	5965	3715	9679	n.m.	0.3	n.m.	0	62	38	0.00	0.62
G5-P	0	5390	2134	7524	n.m.	0.3	n.m.	0	72	28	0.00	0.40
G2-L	0	0	0	0	n.m.	0.3	n.m.	0	0	0	0.00	0.00
G2-P	0	2486	0	2486	n.m.	0.3	n.m.	0	100	0	0.00	0.00
G2-S	0	0	0	0	n.m.	0.3	n.m.	0	0	0	0.00	0.00
G1-S	11,433	43,436	24,016	78,884	1.4	0.4	3.5	14	55	30	0.26	0.55
G1-L	8577	23,094	13,063	44,735	0.7	0.3	2.3	19	52	29	0.37	0.57
G1-P	8161	37,839	21,459	67,458	1.0	0.3	3.3	12	56	32	0.22	0.57
1-KAN S	130,790	40,642	23,783	195,215	1.3	0.3	4.3	67	21	12	3.22	0.59
1-KAN L	24,7895	66,310	29,425	343,630	2.8	0.4	7.0	72	19	9	3.74	0.44
1-KAN P	98,241	55,211	29,562	183,014	2.0	0.3	6.6	54	30	16	1.78	0.54
2-KAN P	12,955	9172	5503	27,631	0.4	0.3	1.3	47	33	20	1.41	0.60
2-KAN S	4967	6430	6916	18,313	0.2	0.3	0.6	27	35	38	0.77	1.08
2-KAN L	14,199	15,955	13,668	43,822	1.1	0.4	2.8	32	36	31	0.89	0.86
W8-S	64,805	182,219	172,801	419,825	4.2	0.4	10.5	15	43	41	0.36	0.95
W8-B	50,083	114,994	104,454	269,531	2.8	0.6	4.6	19	43	39	0.44	0.91
W6-S	38,040	199,426	206,104	443,570	4.0	0.5	8.0	9	45	46	0.19	1.03
W6-B	48,966	221,091	204,843	474,901	5.2	0.5	10.4	10	47	43	0.22	0.93
W5-S	52,847	283,477	277,151	613,475	6.5	0.5	13.0	9	46	45	0.19	0.98
W5-B	60,734	302,348	316,125	679,207	6.3	0.5	12.6	9	45	47	0.20	1.05
W4-B	163,663	481,662	518,632	1,163,957	12.9	0.4	32.3	14	41	45	0.34	1.08
W4-S	182,549	539,768	564,665	1,286,982	13.4	0.4	33.5	14	42	44	0.34	1.05
W3-S	48,530	265,233	273,357	587,121	5.7	0.4	14.3	8	45	47	0.18	1.03
W3-B	54,546	247,411	263,763	565,720	6.0	0.4	15.0	10	44	47	0.22	1.07

^(1)^ Based on LC-HRMS measurements, ^(2)^ based on LC-FLD measurements according to Svenssen et al. [115]. This is only an estimation since it is an indirect method with no standards. n.m. not measured due to lower sensitivity of the HPLC-FLD method. ^(3)^ Conversion to estimated prymnesin content in 1 L of water [110].

**Table 4:** Physical and chemical properties of water in which *P. parvum* was found.

Parameter	Value Range
water temperature	2–30 °C
water clarity	20–70 cm (Secchi disk)
pH	7.2–9.3
salinity	2.2–20‰
alkalinity	5–17.22 meq/L
Cl^−^	339–10,800 mg/L
${SO}_{4}^{-}$	375–7590 mg/L
HCO_3_^−^	0–68.5 mg/L
Ca^++^	45.9–547.6 mg/L
Mg^++^	187.5–905.9 mg/L
Na^+^ and K^+^	75.8–3054.9 mg/L
COD	23.4–42.2 mg/L

Note: COD—chemical oxygen demand [16,95,146].

## Data Availability

Not applicable.
